# Supplementary material for: Using a modular massively parallel reporter assay to discover context-dependent regulatory activity in type 2 diabetes-linked noncoding regions
Source: HGG Adv. 2026 Apr 7;7(3):100606. doi: 10.1016/j.xhgg.2026.100606 (PMC13125165; doi:10.1016/j.xhgg.2026.100606)
Supplement: Document S1. Figures S1–S6 [file mmc1.pdf]

HGGA, Volume 7

Supplemental information

Using a modular massively parallel reporter assay to discover context-dependent regulatory activity in type 2 diabetes-linked noncoding regions

Adelaide Tovar, Yasuhiro Kyono, Kirsten Nishino, Maya Bose, Arushi Varshney, Stephen C. J. Parker, and Jacob O. Kitzman

Supplementary Figures and Figure Legends

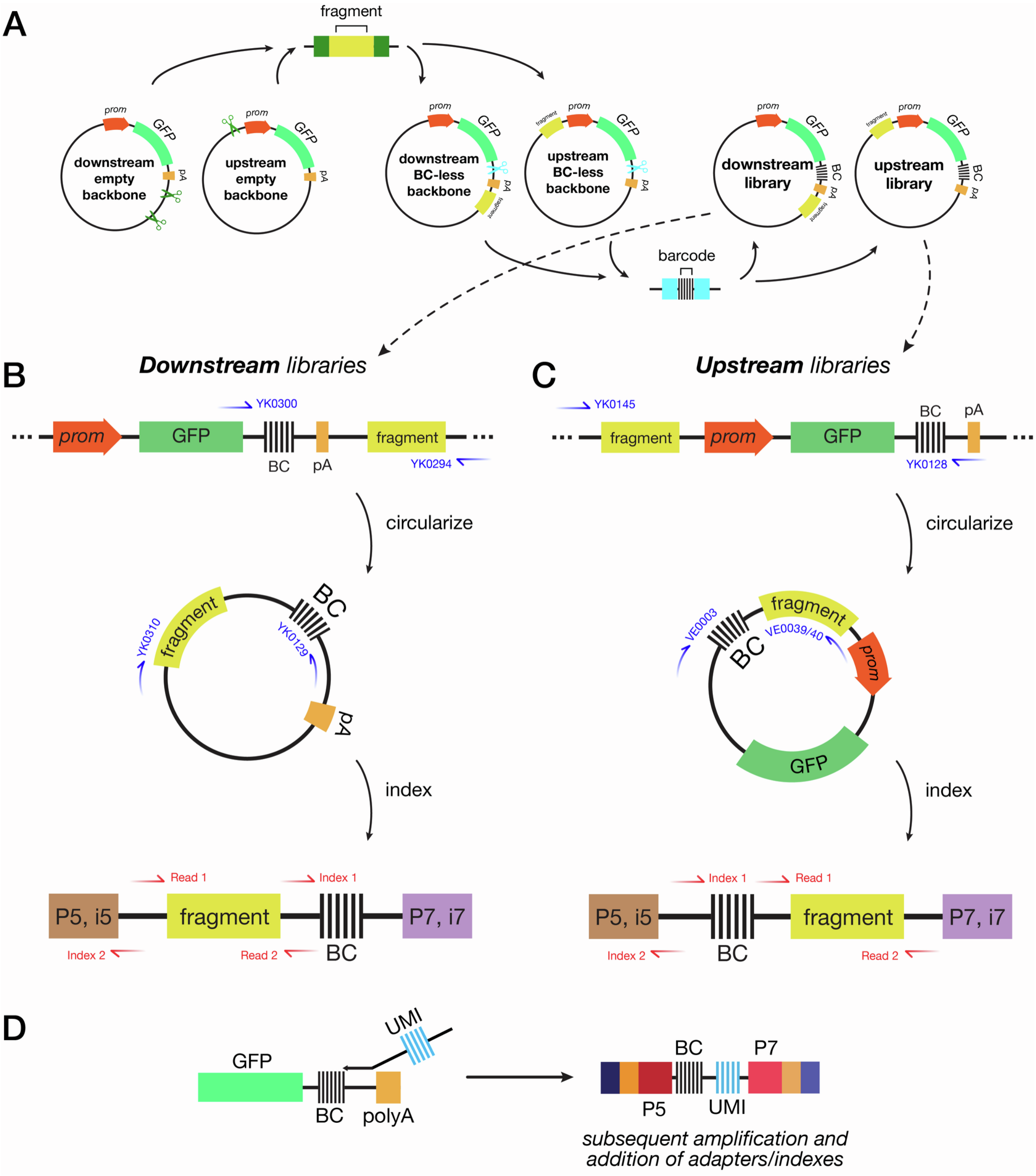

Figure S1. Construction of first MPRA plasmid libraries and barcode sequencing libraries. (A) Construction process for the first MPRA library. We inserted oligo sequences via Gibson assembly into EcoRV- or KpnI-digested (downstream or upstream, respectively) modified STARR-seq backbones that contained either the SCP1 or human insulin (*INS*) promoter. We digested the resulting barcode-less plasmids with PmeI

and inserted barcodes. (B) To perform fragment-barcode pairing for the downstream libraries, we amplified the entire region containing (from 5'-3') the barcode, poly(A) signal, and fragment. We circularized this amplicon and used primers flanking the 5' end of the fragment and 3' end of the barcode. We added sequencing indexes and adapters. We used the index 2 read for sample/library identification, read 1 for the 5' end of the fragment, read 2 for the 3' end of the fragment, and index 1 read for the barcode. **(C)** We performed a similar process as described in **(B)** for the upstream libraries, except the initial amplicon contains (from 5'-3') the fragment, promoter, GFP, and barcode, and the barcode is brought upstream of the fragment after the circularization process. The read layout for the upstream pairing libraries remains the same. **(D)** To construct barcode sequencing libraries using mRNA extracted from cells, we performed reverse transcription using a custom primer containing a unique molecular identifier (UMI) to correct for PCR duplication in sequencing data. Primers were designed separately for each of the two MPRA types to accommodate flanking sequence differences. Subsequently we amplified the cDNA and added sequencing indexes/adapters via PCR.

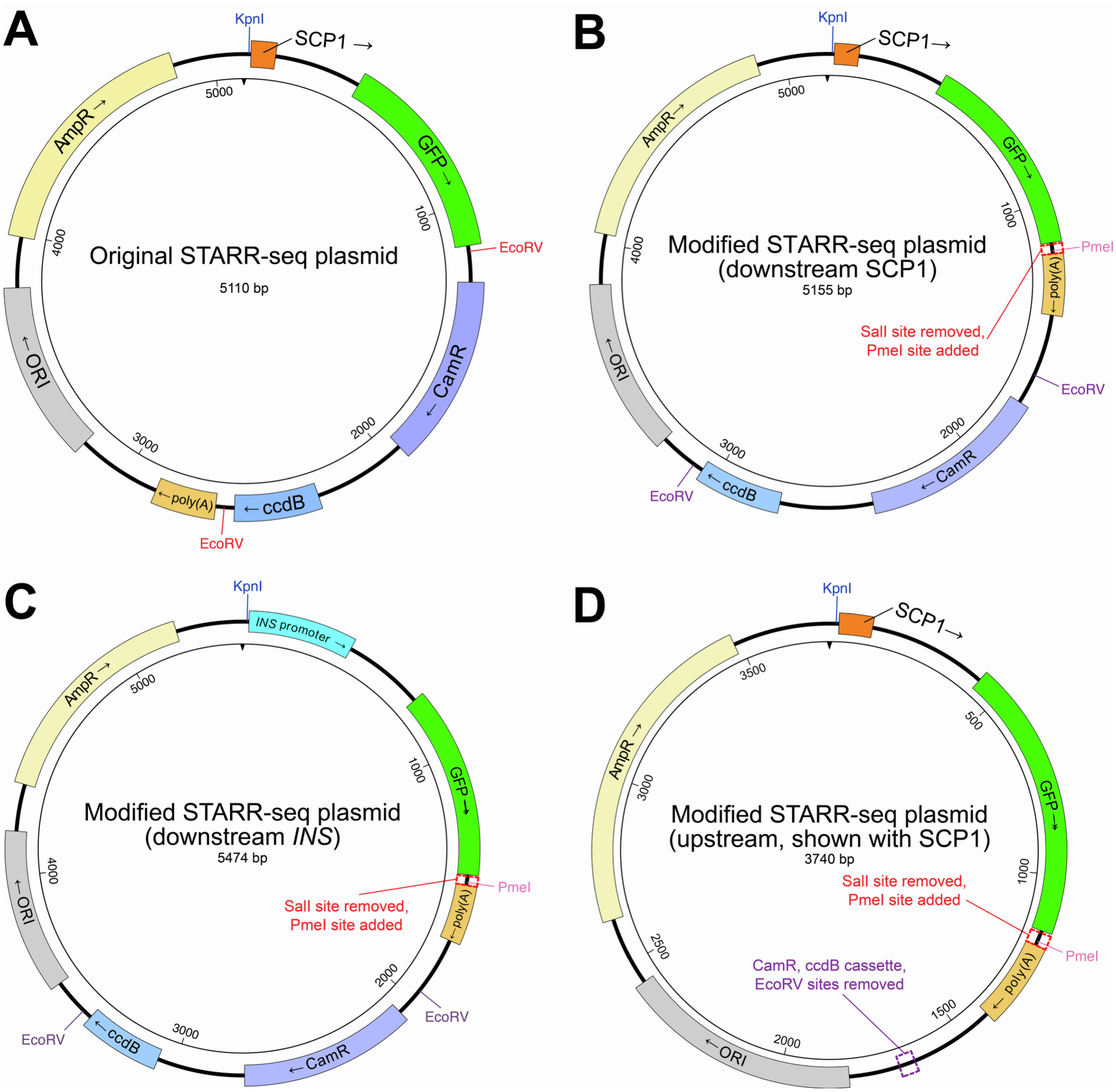

Figure S2. Cloning process to derive modified STARR-seq vectors for the first MPRA library. Arrows next to labels indicate the orientation. **(A)** Simplified diagram for the original STARR-seq vector. **(B)** To generate the downstream SCP1 plasmid, we swapped the position of the ccdB/CamR region and the poly(A) signal while also modifying restriction sites between the 3' end of GFP and the poly(A) signal (removed SalI and added PmeI). Here, the EcoRV restriction sites are used to clone in the fragments. **(C)** We swapped in the *INS* promoter at the SCP1 site to create the downstream *INS* plasmid. **(D)** Finally, to generate the upstream versions of the plasmid we removed the ccdB/CamR region entirely (also removing the EcoRV restriction sites) to bypass the original Gateway system and allow for direct cloning into the vector at the upstream KpnI site.

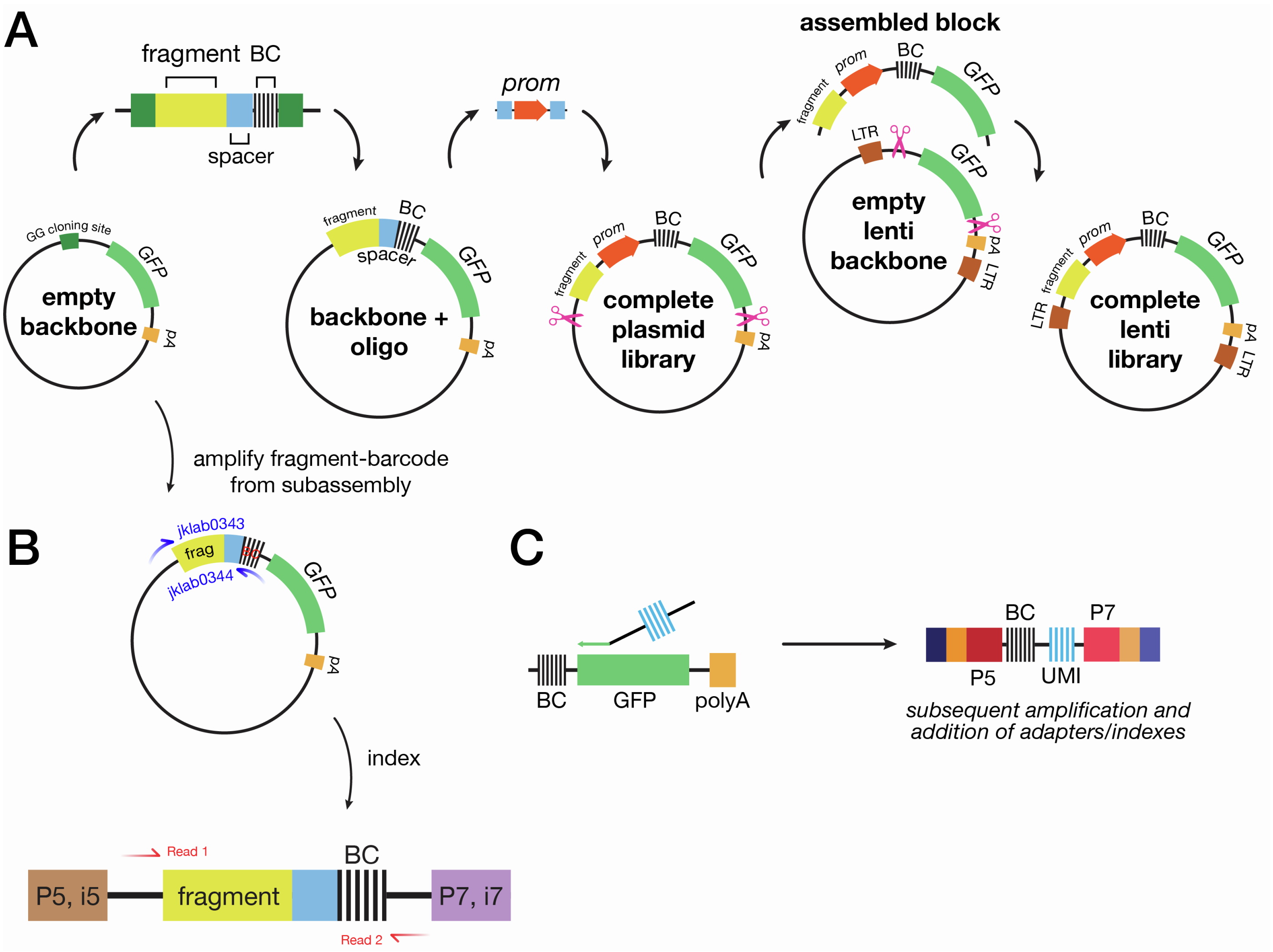

Figure S3. Construction of HNF1 motif-focused MPRA libraries. (A) Construction process for the second MPRA library. We PCR-barcoded oligo sequences and inserted them into the backbone via PaqCI-mediated Golden Gate cloning. We inserted the SCP1 or *INS* promoter in a spacer sequence between the oligo and barcode via BsaI-mediated Golden Gate cloning. **(B)** To construct fragment-barcode pairing libraries, we generated an amplicon spanning the fragment-barcode region of the sub-assembled plasmid. **(C)** Similar to the first MPRA library, we constructed barcode sequencing libraries for the targeted MPRA experiments using a custom primer containing a UMI, this time targeted to the 5' end of GFP.

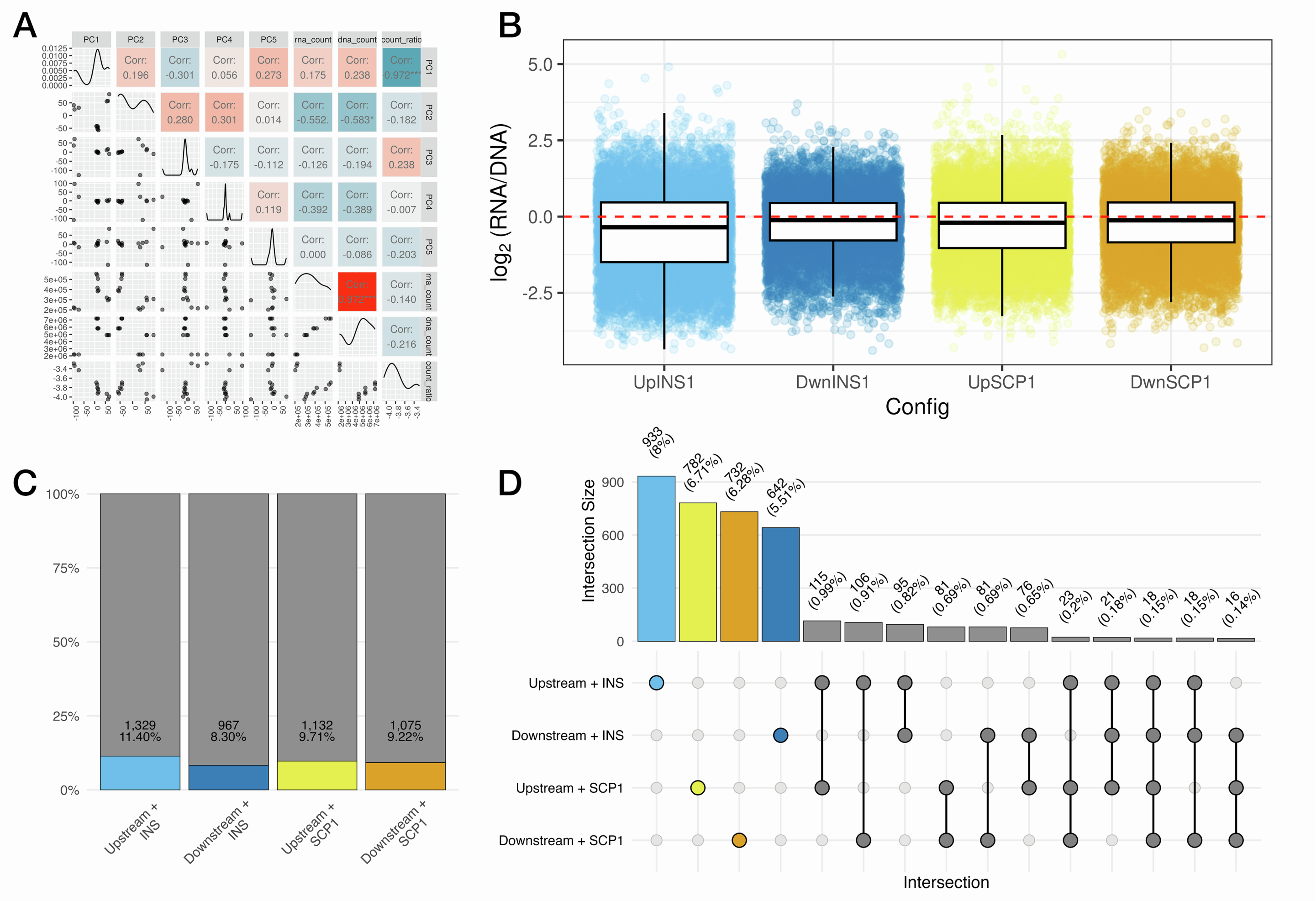

Figure S4. MPRA plasmid configuration modestly influences overall activity of tested fragments and specific sets of significantly active (FDR < 0.05) fragments. (A) Pairwise Spearman correlation heatmap for the first five principal components (from analysis presented in **Figure 2A**), library size-normalized RNA and DNA counts, and ratio between normalized RNA and DNA counts. **(B)** Activity distributions for each of the four configurations shown as the individual activity estimates (average across three replicates). The red dashed line represents a $\log_2$(RNA/DNA) = 0 (i.e., RNA = DNA). (**C)** Stacked barplot showing the number and proportion of fragments that are significantly active (FDR < 0.05) in each plasmid configuration. Percentages are calculated using 11,656 (total number of common fragments) as the denominator. **(D)** UpSet plot displaying intersections (shared and unique) between the sets of fragments that are active in each plasmid configuration. Sets are depicted in the matrix on the bottom, where nodes represent individual configurations (labeled on the left) and edges connect the nodes to form intersections. The bar chart on the top shows the size of each intersection as both the number and proportion of fragments that are significantly active (FDR < 0.05).

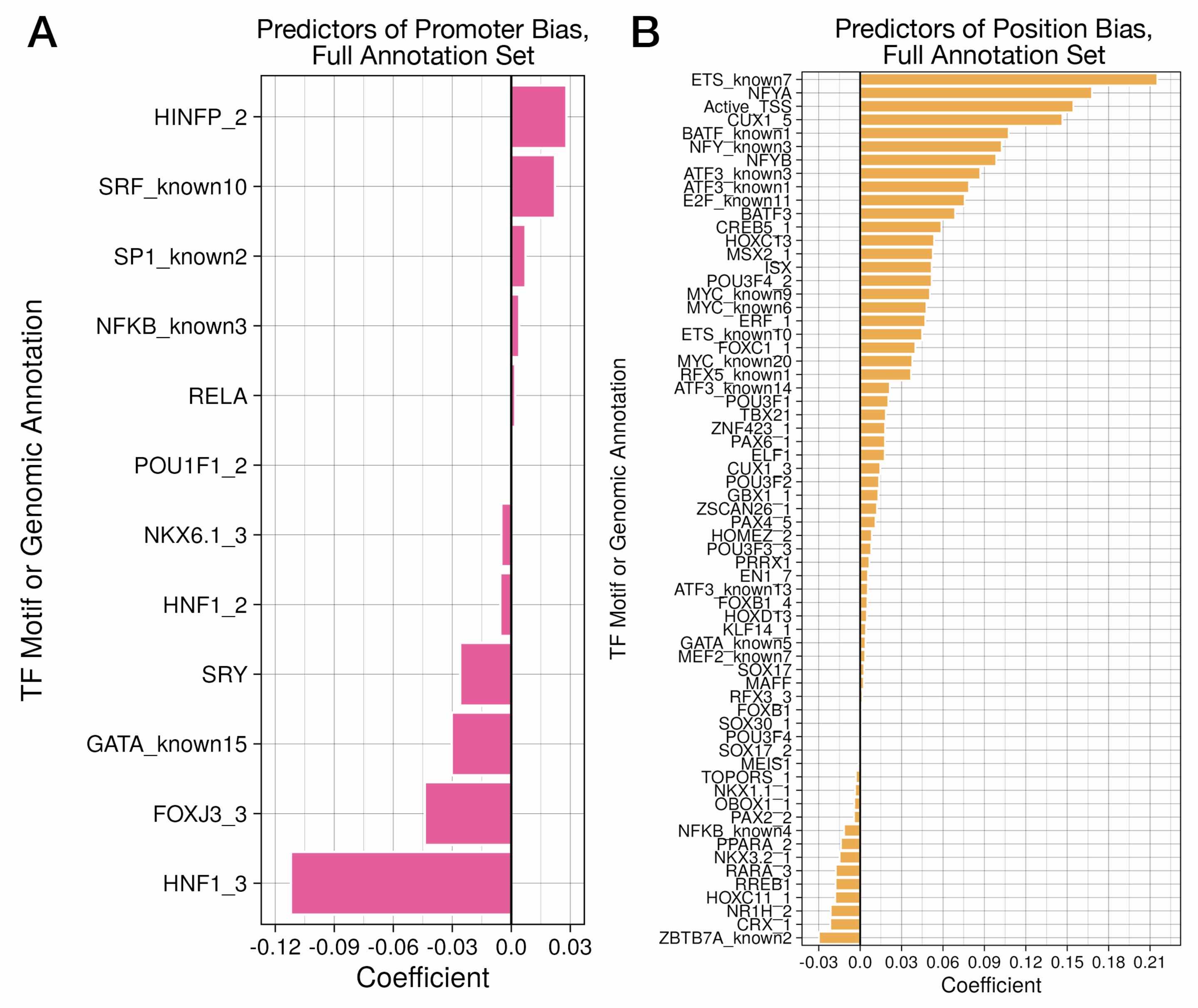

Figure S5. LASSO regression analysis largely identifies the same set of features associated with position or promoter bias when using an expanded set of transcription factor motifs. (A) Full set of significant predictors of promoter bias are displayed as a companion to **Figure 3B**. We identified largely the same set of coefficients. One notable addition is NKX6.1, another beta cell-specific transcription factor. **(B)** Full set of predictors of position bias effects are shown. The 'activeEnhancer' chromatin annotation is no longer listed, though 'X1_Active_TSS' is still among the strongest predictors of upstream positional bias.

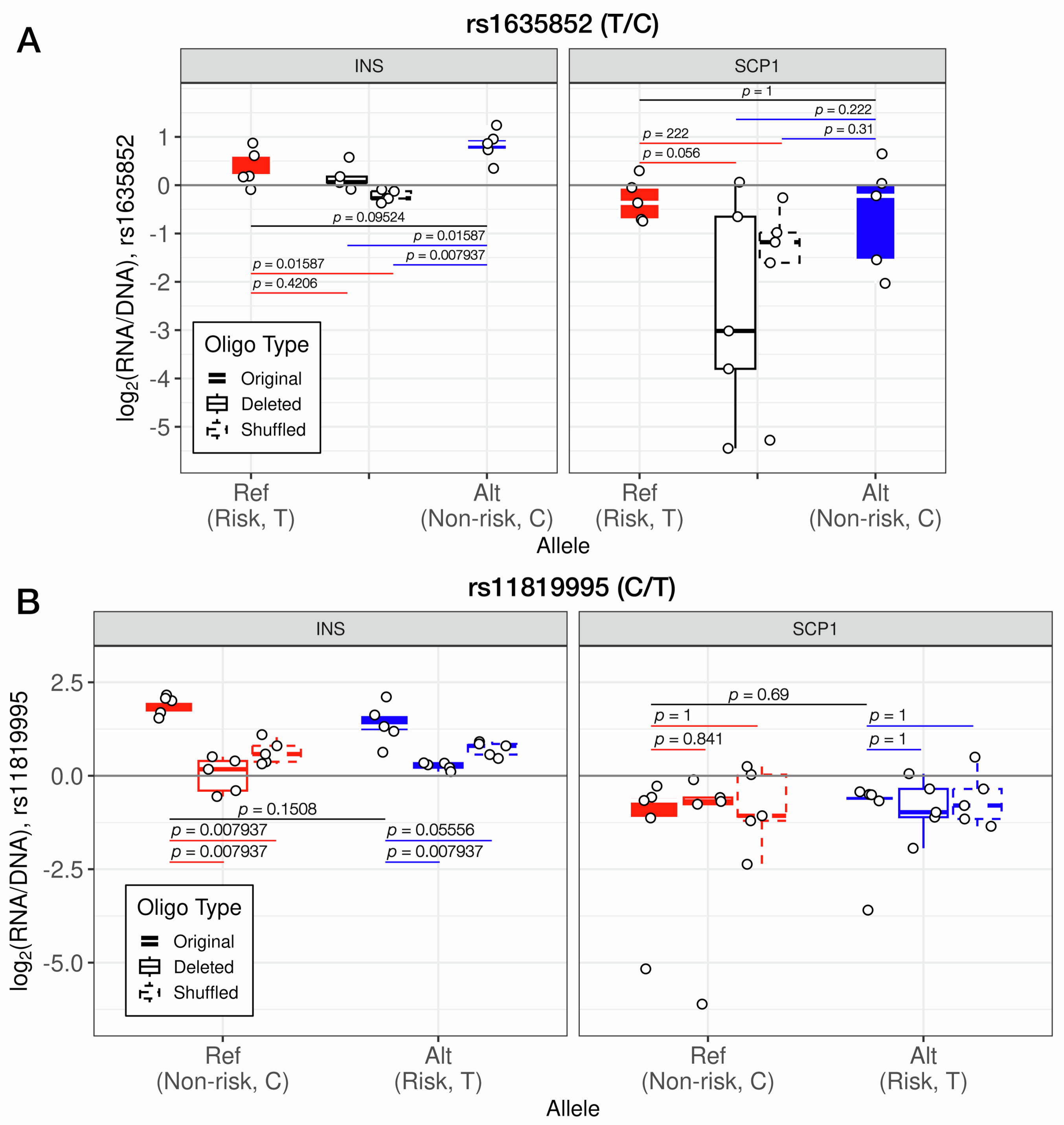

Figure S6. Enhancer activity is minimal for variants near HNF1 transcription factor motifs when paired with the SCP1 promoter. For fragments described in **Figure 4** (reproduced in the left facets), we also examined activity when paired with the synthetic SCP1 housekeeping promoter (displayed in the right facets). **(A)** Manipulating the HNF1 motif overlapping the T2D risk variant rs1635852 does not reproduce the pattern of differential activity seen with the *INS* promoter when paired with the SCP1 promoter, though there is a nearly significant difference in activity between the intact, reference allele oligo and the oligo with the HNF1 motif deleted. We do not observe an allelic difference in activity between the risk (T, reference) and non-risk (C,

alternate) alleles when paired with the SCP1 promoter. **(B)** Similar to the previous variant, none of the fragments for the variant rs11819995 have significant activity when paired with the SCP1 promoter. In contrast to when they're paired with the *INS* promoter, manipulating the HNF1 motif near this variant has no significant effect on activity compared to the original, motif-intact versions of these fragments with the SCP1 promoter (Wilcoxon rank-sum test *p* values reported, n = 5).
