# Supplementary material for: Using a modular massively parallel reporter assay to discover context-dependent regulatory activity in type 2 diabetes-linked noncoding regions
Source: HGG Adv. 2026 Apr 7;7(3):100606. doi: 10.1016/j.xhgg.2026.100606 (PMC13125165; doi:10.1016/j.xhgg.2026.100606)
Supplement: Document S2. Article plus supplemental information [file mmc17.pdf]

HGG
Advances

ARTICLE

Using a modular massively parallel reporter assay to discover context-dependent regulatory activity in type 2 diabetes-linked noncoding regions

Adelaide Tovar,[1] Yasuhiro Kyono,[1,4] Kirsten Nishino,[1] Maya Bose,[1] Arushi Varshney,[1] Stephen C.J. Parker,[1,2,3,*] and Jacob O. Kitzman[1,2,5,*]

Summary

Most complex trait association signals reside in the noncoding genome, where defining function is challenging. MPRAs (massively parallel reporter assays) offer a scalable means to test variants' regulatory impacts but are typically cell-type agnostic, pairing cloned fragments with generic "housekeeping" promoters. To explore MPRAs' context sensitivity, we screened a panel of nearly 12,000 fragments across >300 diabetes- and metabolic-trait-associated regions in a pancreatic β cell line model. We compared activity when fragments were placed up- versus downstream of a reporter gene and combined with the synthetic housekeeping promoter super core promoter 1 (SCP1) versus the physiologically relevant human insulin (*INS*) gene promoter. We identified clear effects of MPRA construct design on regulatory activity. A subset of fragments ($n = 702/11,656$) displayed positional bias, evenly distributed across up- and downstream preferences. Promoter choice also influenced MPRA activity ($n = 698/11,656$), mostly biased toward the cell-specific *INS* promoter (73.4%). A screen for sequence annotations associated with *INS* promoter preference revealed enrichment for HNF1 binding motifs. HNF1 family transcription factors are key regulators of glucose metabolism disrupted in maturity-onset diabetes of the young (MODY), suggesting genetic convergence between rare coding variants that cause MODY and common type 2 diabetes (T2D)-associated regulatory regions. A follow-up HNF1-focused MPRA highlighted several instances where motif deletion or mutation disrupted regulatory activity specifically in the context of the *INS1* promoter and in the β cell model but not in another diabetes-relevant cell type, skeletal muscle. These results identify technical factors that may require careful consideration while designing MPRA experiments.

Introduction

Complex diseases arise from individual and interactive effects of genetic, environmental, and lifestyle factors. Recent genome-wide association studies (GWASs) have revealed that the majority of complex disease-associated loci (~90%) are found in noncoding regions where they are expected to alter gene regulation.[1] Progress to identify causal variants and define their role(s) in disease pathogenesis has been slow due to the lack of a clear variant-to-function framework for noncoding variants. Massively parallel reporter assays (MPRAs) have emerged as a useful method for screening large collections of putative regulatory elements, learning principles of disease-associated regulatory variation, and efficiently prioritizing candidate causal variants at disease loci.[2–5]

A wide array of MPRA designs exists, primarily differing by the position where the fragments of interest are cloned relative to a basal promoter and by the reporter gene (summarized in Klein et al.[6]). Enhancers are classically defined as acting at a distance in an orientation-independent manner,[7] though how distinct they are from promoters remains unclear. Klein et al. reported modest impacts of fragment position on enhancer activity, which could nevertheless mask the often subtle regulatory effects being measured,[3,8–12] clouding downstream interpretation.

In addition to fragment positioning, another key design consideration is the choice of promoter. Ideally, regulatory variants would be tested in the context of their cognate endogenous promoters; however, target genes remain unknown at most disease-associated signals. MPRAs bypass this barrier by measuring regulatory activity independent of a tested element's target gene. To date, most MPRAs used to study disease-associated genetic variants rely on a standard design featuring one of several housekeeping promoters (e.g., minimal promoter [minP] and super core promoter 1 [SCP1]).[3,13–15] There is conflicting evidence as to the influence of promoter-enhancer compatibility rules: some studies suggest that unique sequence features, including transcription factor (TF) motifs, dictate how strongly an interaction will occur,[16,17] while others indicate that there is minimal specificity with a simple additive model combining intrinsic enhancer and promoter strength.[5,18,19] There have been limited comparisons of MPRA design parameters, including fragment position and enhancer-promoter compatibility, and how these

[1]Gilbert S. Omenn Department of Computational Medicine and Bioinformatics, University of Michigan, Ann Arbor, MI 48109, USA; [2]Department of Human Genetics, University of Michigan, Ann Arbor, MI 48109, USA; [3]Department of Biostatistics, University of Michigan, Ann Arbor, MI 48109, USA
[4]Present address: Seattle Hub for Synthetic Biology, Allen Institute, Seattle, WA 98109, USA
[5]Lead contact
*Correspondence: scjp@umich.edu (S.C.J.P.), kitzmanj@umich.edu (J.O.K.)
https://doi.org/10.1016/j.xhgg.2026.100606.
© 2026 The Authors. Published by Elsevier Inc. on behalf of American Society of Human Genetics.
This is an open access article under the CC BY license (http://creativecommons.org/licenses/by/4.0/).

choices interact in disease-relevant cell types has not yet been systematically examined. We speculated that using a promoter active in the appropriate cell type might better capture cell-type-specific regulatory mechanisms than a generic minP.

Here, we used a modular MPRA to assess the regulatory activity of genomic regions overlapping type 2 diabetes (T2D)-associated signals across two promoter and two position contexts, allowing us to evaluate how MPRA design choices influence the detection of active regulatory elements.

Material and methods

Cell culture

We obtained INS-1 832/13 rat insulinoma cells from Dr. Christopher Newgard (Sarah W. Stedman Nutrition and Metabolism Center, Duke University, Durham, NC) and LHCN-M2 human skeletal muscle myoblasts from Evercyte. We cultured 832/13 cells in RPMI-1640 containing 10% fetal bovine serum, 0.05 mM β-mercaptoethanol, 1 mM sodium pyruvate, 2 mM L-glutamine, 10 mM HEPES, and 1,000 U/mL penicillin/streptomycin. We maintained LHCN-M2 cells on 0.1% gelatin-coated flasks in 4:1 DMEM (4.5 g/L glucose, glutamine, and bicarbonate):Medium 199 (with bicarbonate) containing 15% FBS, 20 mM HEPES, 30 ng/mL zinc sulfate, 1.4 μg/mL vitamin B12, 55 ng/mL dexamethasone, 2.5 ng/mL recombinant human hepatocyte growth factor, 10 ng/mL basic fibroblast growth factor, and 600 U/mL penicillin/streptomycin.

Large (first) MPRA library design, construction, and delivery

We compiled regions of interest for the large MPRA library from loci associated with T2D and related metabolic traits, drawing on signal variants reported by the DIAMANTE Consortium[20] and signal variants for HbA1C, fasting insulin adjusted for BMI, fasting glucose adjusted for BMI, and 2-h glucose adjusted for BMI reported by the MAGIC Consortium.[21] To provide a broader disease context, we also included loci for non-T2D traits, including rheumatoid arthritis, systemic lupus erythematosus, and type 1 diabetes. We used UK Biobank imputed genotypes to calculate linkage disequilibrium and fetch proxies with an R^2 greater than 0.8. We removed any variants that were not SNVs (i.e., insertions or deletions [indels] and copy-number variants [CNVs]), resulting in a set of 13,226 sites (Table S1). For each site, we designed 198-bp fragment sequences in three positional configurations relative to the focal base: offset left (position 50 of 198 bp), centered (position 99), or offset right (position 149). In cases where a variant had two alternative alleles, we designed both sets of sequences. We extracted all sequences from the hg19 reference genome. We added 16-bp flanking adapter sequences for PCR amplification and cloning. We obtained a pool of 79,380 full-length 230-bp fragments from Agilent.

We constructed the large MPRA library as described previously[22] (Figure S1A). We first generated four sub-cloned plasmids derived from the original STARR-seq vector[4] (Figure S2A). For the first vector (downstream SCP1), we swapped the positions of the ccdB survival cassette and SV40 poly(A) signal to bring the latter closer to the 3′ end of GFP and added a PmeI site between the 3′ end of GFP and poly(A) signal to enable libraries to be post-barcoded via Gibson assembly (Figure S2B). The second vector was further derived from the first vector (downstream insulin [*INS*] promoter) by swapping out the SCP1 promoter for the 408-bp *INS* promoter (Figure S2C). Finally, the third and fourth vectors were used for the upstream libraries and were derived from their respective downstream promoter vector (Figure S2D). We removed the entire ccdB cassette and, with it, the EcoRV restriction sites used to clone the fragment in the downstream position, leaving only the KpnI site used for upstream cloning.

To prepare the fragments for cloning, we used PCR to add homology arms, using distinct primer pairs for three separate sub-pools within the full Agilent pool (complete pairing details in Table S2). We pooled the PCR products proportional to the number of oligos in each sub-pool and used Gibson assembly to clone them into a KpnI- or EcoRV-digested (upstream or downstream, respectively) modified STARR-seq backbone (Figure S1A). We transformed these first vectors containing all components without barcodes into NEB 10-beta electrocompetent cells. We post-barcoded the first vectors using Gibson assembly to insert 16-bp random barcodes at the PmeI restriction site. We transformed this reaction into electrocompetent 10-beta cells and prepared final plasmid libraries for transfection using the ZymoPURE Plasmid Maxiprep Kit.

To pair barcodes with their corresponding fragments (Figures S1B and S1C), we used PCR to generate one of two amplicons. For the downstream libraries, where the barcode and fragment are separated only by the poly(A) signal, we used a forward primer binding immediately upstream of the barcode and a reverse primer immediately downstream of the fragment. For the upstream libraries, where the barcode and fragment are separated by the promoter, GFP, and poly(A) signal, we used a forward primer binding immediately upstream of the fragment and a reverse primer binding immediately downstream of the barcode. For both amplicon types, we phosphorylated and self-ligated the products to form circularized fragments to bring the fragment and barcode next to each other, then used Plasmid-Safe ATP-Dependent DNase to selectively deplete linear fragments. Finally, we used primers to amplify across the barcode and fragment and then indexed samples with custom primers.

For the first library, we electroporated 50 μg of plasmid into 25 million 832/13 rat insulinoma cells for each biological replicate. While we did not measure electroporation efficiency for this experiment, we used an electroporation protocol that has been optimized for 832/13 cells and generally yields 50%–60% efficiency. We collected and lysed cells with TRIzol 24 h later. After phase separation, we used the Direct-zol RNA Miniprep Kit to isolate total RNA. We enriched for mature mRNA transcripts with oligo(dT) beads, then treated 2 μg of mRNA with DNase I to remove plasmid or genomic DNA contamination. We reverse transcribed 1 μg of mRNA using SuperScript III with a custom primer containing a 6-bp unique molecular identifier (UMI) targeting transcripts derived from the MPRA plasmids (Figure S1D). We treated cDNA samples with DpnI to eliminate any residual plasmid DNA. Separately, we used Illumina-compatible primers to create indexed sequencing libraries for the input plasmid DNA.

All primers used to generate the MPRA library, fragment-barcode pairing libraries, and barcode counting libraries are listed in Table S2.

Motif (second) MPRA library design, construction, and delivery

We constructed the motif MPRA library using a modified version of the methods detailed in Tewhey et al.[3] While the cloning strategy differed slightly from what we used for the first library, the assay design and functional readout between the two are comparable (Figure S3A). To enable library delivery to cell types, we devised a strategy to construct paired MPRA libraries in both a plasmid backbone and a lentiviral transfer vector (i.e., the barcode-fragment associations are constant across systems). Given the known consequences of lentiviral template switching on fragment-barcode associations,[23,24] we chose to clone all fragments in the upstream position with the barcode placed between the promoter and GFP.

We selected fragment sequences from the first library with strong *INS*-promoter-biased activity (false discovery rate [FDR] < 0.05) that overlapped one or more HNF1 motifs, without imposing a position-bias activity cutoff. We obtained fragments spanning the same amount of genomic sequence as the previous library but containing different adapters (IDT). We modified the pMPRA1 vector (a gift from Tarjei Mikkelsen; Addgene #49349) by removing the ccdB survival cassette and adding in a second PaqCI restriction site. We added barcodes and a promoter-cloning scaffold to fragments via PCR, then cloned the amplified fragments into our modified vector using Golden Gate assembly with PaqCI. We digested this assembly with SfiI to remove empty backbones, then transformed it into electrocompetent 10-beta cells. We used this assembly to perform fragment-barcode association by generating a PCR product spanning the full fragment and barcode window of the plasmid (Figure S3B). To create the final, complete plasmid library, we inserted clonal promoter fragments using Golden Gate assembly with BsaI and digested with AsiSI to remove promoterless constructs. We transformed this final assembly into electrocompetent 10-beta cells, expanded in 150 mL cultures, and isolated the plasmid using the ZymoPURE Plasmid Maxiprep Kit. For use with the LHCN-M2 skeletal muscle myocyte cell line, we ported the assembled MPRA block (fragment, barcode, promoter, and GFP) to a lentiviral transfer vector (a gift from Nadav Ahituv; Addgene #137725) via restriction cloning.[25,26]

For the second library, we electroporated 30 μg of plasmid into 30 million 832/13 cells. We lysed cells 24 h later with Buffer RLT Plus and isolated total RNA with the Qiagen RNeasy Midi Kit. For all samples generated through the use of the second set of MPRA libraries, we reverse transcribed 30 μg of DNase I-treated total RNA into cDNA with a GFP-specific primer containing a 6-bp UMI (Figure S3C), then constructed indexed sequencing libraries for both the cDNA and input (plasmid or gDNA) libraries using Illumina-compatible primers (Table S3).

The University of Michigan Viral Vector Core produced infectious lentiviral particles with this transfer vector and third-generation lentiviral plasmids in HEK293T cells. Per replicate, we infected 4×10^6 LHCN-M2 human skeletal myoblasts with our MPRA library at an MOI of ~10, to achieve an average of 10 integrations per barcode. After infection, we passaged the cells for 1 week to remove any unincorporated virus or contaminating transfer plasmid, then differentiated the cells for 1 week. We isolated RNA and gDNA from each replicate using the Qiagen AllPrep DNA/RNA mini kit.

Sequencing data collection

We sequenced pairing (with custom primers) and cDNA/input sequencing libraries for the initial set of MPRA studies on an Illumina HiSeq system. We sequenced libraries generated for the second set of MPRAs on an Illumina NovaSeq 6000 with standard primers. We received pairing library data across three reads for the first MPRA (Figures S1B and S1C). We received paired-end 150-bp reads for the barcode-counting libraries for both the first and second MPRAs and for the pairing libraries of the second MPRA.

To create the barcode-fragment pairing dictionary, we used a custom pipeline.[27] First, we used starcode to cluster barcode groups to generate a full set of all possible barcodes.[28] Next, we merged and aligned the paired-end 150-bp reads spanning the fragment against our reference FASTA file using bwa v.0.7.17.[29] Finally, we used freebayes to identify sequencing or synthesis errors in the aligned fragments, then filtered all fragment-barcode pairs for mapping quality and sequencing depth.[30] After removing any duplicate barcodes, we created a final table with fragment-barcode pairs.

We processed MPRA sequencing data using a custom pipeline that quantifies barcode counts while accounting for possible sequencing errors. We identified reads with the barcode sequence and trimmed constant sequences using cutadapt v.4.3.[31] We used UMI-tools v.1.1.2 to identify and cluster UMIs, then used starcode-umi to cluster UMI-barcode and perform deduplication.[28,32] Finally, we used starcode to cluster and count barcodes.

Activity estimation and joint modeling

Prior to model fitting, we merged cDNA and input barcode counts with the corresponding pairing dictionary, retaining only exact barcode sequence matches. For both MPRA libraries, we used the R package MPRAnalyze to estimate regulatory activity and promoter or position bias.[33] This modeling approach fits two separate models for cDNA and input counts, then relates the two to yield a per-fragment transcription rate. For all analyses, resulting regulatory activity scores were converted from a natural log scale to a binary log scale, although values are reported on both scales in Tables S4, S5, S6, S7, and S13.

For the first MPRA library, we used this method to estimate regulatory activity within each construct type and then performed post hoc comparisons outside of the analysis framework. To evaluate the quantitative effects of promoter and position on fragment activity in the first MPRA library, we jointly estimated regulatory activity across all four constructs and included promoter and position as covariates. While we did originally design the library with variants in offset left, center, and offset right positions within the fragment, we did not evaluate within-fragment variant positioning effects owing to the technical limitations described in the results section.

For the second MPRA library, we used this method to estimate regulatory activity across the entire library in 832/13 cells and nominate candidate fragments for further inspection. Because the second MPRA library contained negative controls, we used this reference set for activity testing within the MPRAnalyze framework. Then, on a per-fragment basis, we compared activity differences across fragment types using a Wilcoxon rank-sum test. For visualization purposes, we normalized activity estimates to negative controls.

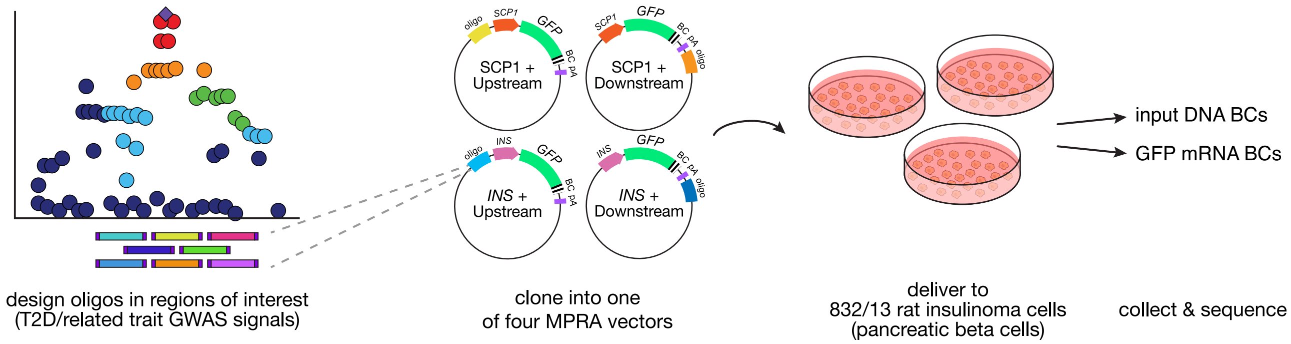

Figure 1. Study design
We synthesized a library of 198-bp fragments encompassing 13,226 sites in high linkage disequilibrium with type 2 diabetes (T2D) and related trait-associated signals. We cloned this library into each of four MPRA constructs: fragment upstream or downstream of the human insulin (*INS*) promoter or upstream or downstream of the super core promoter (SCP1). We simultaneously delivered all four libraries to 832/13 rat insulinoma cells ($n = 3$) and collected DNA and RNA for sequencing.

LASSO regression to select features associated with promoter or position bias

Here, we used least absolute shrinkage and selection operator (LASSO) regression to model MPRA activity bias (promoter or position) and select genomic features associated with this bias.[34] LASSO regression uses a penalty term to shrink coefficients of less informative variables to zero, thereby removing them from the model and enhancing interpretability. We quantified activity bias by using the Wald statistics from our joint estimates of regulatory activity and multiplying them by the sign of the log fold change to indicate the direction of bias (INS versus SCP1 for promoter and up- versus downstream for position). As features in our models, we used a set of annotations and TF motifs described previously.[22,35] For each fragment, we quantified motif occurrences or annotation overlap. To generate overlap scores that could be used as parameters for LASSO regression and compared uniformly across annotations, we inverse normalized the $-\log_{10}(p \text{ value})$ for each motif or annotation overlap using R/RNOmni.[36] We ran the regression using R/glmnet with default parameters ($\alpha = 1$ corresponding to LASSO) and 10-fold cross-validation using the signed Wald statistic as the outcome and annotation scores as the predictors.[37] We performed regression analyses for the promoter and position bias scores separately, and glmnet automatically determined the appropriate lambda value by minimizing the mean cross-validated error.

Results

Massively parallel reporter design and cloning

To explore regulatory activity across loci associated with T2D and related metabolic traits, we designed a library of 13,226 targets spanning regions in high linkage disequilibrium ($R^2 > 0.8$) with 696 index signals reported by the DIAMANTE[20,38,39] and MAGIC[21] consortia (Figure 1; Table S1). We added short 16-bp anchor sequences on both sides of each fragment to enable cloning into the MPRA constructs. Using a two-step process, we cloned this fragment library into four different MPRA constructs: upstream or downstream of a portion of the human insulin (*INS*) promoter or the synthetic super core promoter (SCP1)[40] (Figures S1A–S1C). At each step, transformant pools were maintained at ~1.5 million clones, yielding an average of ~20 barcodes per fragment. We simultaneously delivered the resulting plasmid libraries to the 832/13 rat insulinoma cell line via electroporation, then collected cells 24 h later to isolate RNA. We performed this experiment in triplicate and prepared barcode sequencing libraries from the cDNA and plasmid input to estimate enhancer activity of each fragment (Figure S1D).

Regulatory activities measured across fragments

Our MPRA library contained both reference and alternative alleles but was not barcoded to sufficient complexity for allelic effect detection. Accordingly, we focused on patterns of regulatory activity across sequence contexts rather than allelic bias in activity. After filtering for fragments represented by greater than two barcodes and with at least ten input DNA counts across all four configurations, we proceeded with a set of 11,656 fragments for downstream analysis. This set of fragments represents 6,172 unique sites in high linkage disequilibrium with 600 GWAS signal variants.

To qualitatively examine the influence of position and promoter on fragment activity, we performed principal-component analysis (PCA; Figure 2A) on $\log_2$(RNA/DNA) activity estimates using library size-normalized counts. We identified that the four constructs separated on principal component 2 (PC2; 13.25% of variance) based on the fragment position, with both downstream constructs clustering together, separate from the two upstream constructs. The two upstream constructs further separated on PC1 (15.68% of variance) based on the promoter used. To evaluate whether other technical factors confounded the group separations we saw using PCA, we calculated pairwise Spearman correlation between the first five PCs, RNA library size, DNA library size, and ratio of RNA library size/DNA library size (library size ratio) (Figure S4A). We identified a strong negative correlation ($R^2 = 0.972$) between PC1 and library size ratio, suggesting a relationship between PC1 and overall regulatory activity for a given library. Additionally, we

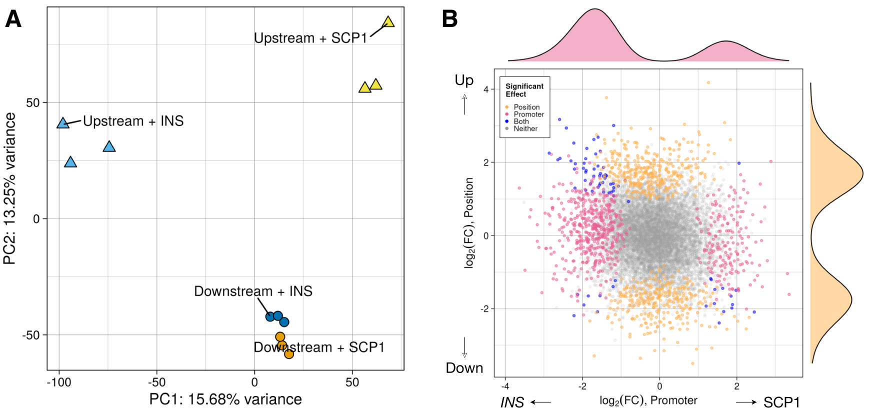

Figure 2. Promoter and fragment position relative to the promoter and reporter gene influence MPRA activity
(A) After fragment activity quantification in R/MPRAnalyze, we performed principal-component analysis to estimate the broad influence of promoter (captured primarily by PC1) and position (captured by PC2) on MPRA activity. Each point represents an individual replicate; points are color coded based on plasmid configuration, and point shape indicates the position of the cloned fragment.
(B) To jointly estimate effects of promoter and position across fragments in all four configurations, we included promoter and position as covariates in the MPRAnalyze model. This scatterplot displays the coefficients from Wald testing for the promoter (pink points along the x axis, *INS* promoter versus SCP1 promoter with local FDR < 0.05) and position (orange points along the y axis, upstream versus downstream with local FDR < 0.05). Marginal histograms displayed above and to the right of the scatterplot display effect size distributions for fragments with significant promoter or position effects. PC, principal component; FC, fold change

observed a strong relationship between RNA library size and DNA library size ($R^2 = 0.972$), which is expected given that RNA counts are partially dependent on plasmid DNA counts. Otherwise, we observed no strong correlations between any of the PCs and technical factors.

To systematically test all fragments in each library for regulatory activity, we used the R package MPRAnalyze,[33] which estimates activity by comparing the RNA counts for each barcode to DNA counts for the same barcodes (Tables S4, S5, S6, and S7). Overall differences in regulatory activity between the four configurations (Figure S4B) largely aligned with the proportion of fragments categorized as significantly active by MPRAnalyze (Figure S4C; FDR < 0.05 and mean $\log_2$(RNA/DNA) > 1): 1,329 (of 11,656 total fragments tested, 11.40%) downstream of the *INS* promoter, 967 (8.30%) upstream of the *INS* promoter, 1,132 (9.71%) downstream of the SCP1 promoter, and 1,075 (9.22%) upstream of the SCP1 promoter. These proportions are comparable to those calculated when using all fragments without requiring their representation across all four configurations (Table S8). Of the set of 11,656 fragments, only 18 (0.15%) were significantly active across all four configurations (Figure S4D). We also defined sets of fragments that were uniquely active in a single configuration, representing a collective 3,089 fragments (26.50%): 933 (8%) upstream of the *INS* promoter, 782 (6.71%) upstream of the SCP1 promoter, 732 (6.28%) downstream of the SCP1 promoter, and 642 (5.51%) downstream of the *INS* promoter (Figure S4D). Taken together, these initial analyses indicate that each MPRA construct influences measured regulatory activity in a unique manner, where most fragments were significantly active across only one or two constructs of the four tested.

Quantifying promoter and position effects

We systematically quantified the effects of each promoter and position on an individual fragment basis using a linear modeling approach in MPRAnalyze. We modeled the activity of each fragment with position and promoter as covariates, then performed a Wald test between each contrast (SCP1 versus *INS* and upstream versus downstream) to extract model coefficients (Tables S9 and S10). Overall, we identified 698/11,656 fragments (5.99%) with significant promoter effects (Figure 2B; FDR < 0.05) and 703/11,656 fragments (6.03%) with significant upstream/downstream position effects. Between the two groups, only 74 fragments display both promoter and position effects. While fragments with significant position effects were roughly divided between up- and downstream bias, we observed that 73.35% of fragments with significant promoter effects were biased toward the *INS* promoter ($n = 512/698$; $p = 2.2 \times 10^{-16}$, binomial test with 55% expectation, or the proportion of fragments active with the *INS* promoter). The sequence content of the SCP1 promoter is markedly different from the *INS* promoter used here. SCP1 is an 81-bp sequence composed of four highly conserved core promoter motifs[40]: a TATA box, the initiator element (Inr), a motif ten element (MTE), and the downstream promoter element (DPE). SCP1 is known to act as a strong promoter for enhancers in conventional reporter constructs and was used in one of the first genome-wide pooled reporter assays, STARR-seq.[4] By contrast, we used a 408-bp fragment of the *INS* promoter spanning −364 to +44 bp of the human *INS* gene,[41] which contains a battery of pancreas-expressed TF binding sites (e.g., RFX6, NEUROD1, and INSM1)[42] and designated glucose-responsive elements.[43] We hypothesized that specific sequence features of the *INS* promoter may enable regulatory compatibility with a broader subset of diabetes and related trait-associated fragments.

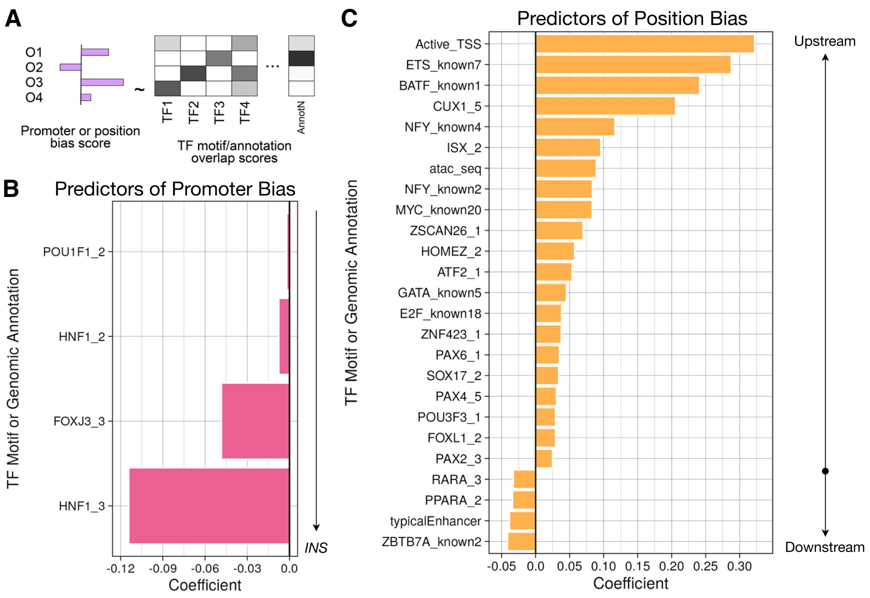

Figure 3. Position and promoter bias in enhancer activity are partially attributed to the presence of relevant annotations

(A) To select sequence features associated with position or promoter bias, we performed LASSO regression with 10-fold cross-validation on position- or promoter-bias scores (signed Wald statistics) as a function of several thousand transcription factor motifs and genomic annotations.

(B) Significant predictors of promoter bias are displayed. With the clustered set of annotations, we identified only features enriched in fragments that are more active with the *INS* promoter.

(C) Top 25 predictors (i.e., non-zero coefficients) associated with position-bias effects are shown. Positive coefficients indicate annotations that are enriched in fragments that are more active in the upstream position, while negative coefficients are enriched in fragments that are more active in the downstream position.

Sequence features associated with position or promoter bias

Next, we were interested in defining specific sequence features associated with promoter- or position-driven activity bias using a set of genomic annotations, including TF motifs, accessible chromatin peaks measured in human pancreatic islets, and ChromHMM-based chromatin state inferences derived from genomic profiling in islets (Figure 3A). Given that this set of annotations is large (~3,000 features), we anticipate that only a small subset is associated with activity bias. To address this challenge, we used LASSO regression, an extension of linear regression that penalizes and thereby removes uninformative predictors, resulting in a simple, interpretable model. For each unique fragment sequence, we calculated overlap scores across all annotations, which we standardized for use as model predictors via rank-based inverse normalization. We performed LASSO regression with 10-fold cross-validation, where the annotation overlap scores were the predictors and the position- or promoter-bias scores (i.e., signed Wald statistics from the previous joint analysis) were the outcome variables. To account for redundancy in TF motifs, we first performed LASSO using a clustered set of 540 motifs[35] and compared the results with those obtained using the full set of annotations (Tables S11, S12, S13, and S14). Only four features showed significant association with promoter bias, with all four enriched in fragments with *INS*-promoter-biased activity versus SCP1 (Figure 3B). Notably, two of these were motifs for HNF family TFs, which play important roles in pancreatic β cell development, differentiation, and homeostasis and, when disrupted, cause maturity-onset diabetes of the young (MODY), an early-onset monogenic form of diabetes.[44,45] Concordantly, many noncoding variants associated with complex, later-onset diabetes (i.e., T2D) are enriched in HNF1 motifs.[46] Additionally, the *INS* promoter itself overlaps a putative HNF1 motif; however, there is conflicting evidence as to whether HNF1A/HNF1B directly mediates *INS* transcription.[47–49] Together, this regulatory convergence suggests that these two TFs serve as critical regulators of transcriptional activity central to diabetes pathogenesis.

When performing LASSO with the unclustered set of motifs, we additionally found that motifs for NF-κB constituents (RELA/p65 and NFKB) and the ubiquitously expressed activator TFs SP1 and SRF were associated with SCP1-promoter-biased fragment regulatory activity (Figure S5A). SP1 functions by interacting with the TF II D complex ($TF_{II}D$),[50] a constituent of the RNA polymerase II preinitiation complex that binds to core sequences found in the SCP1 promoter.[51] HINFP (also known as MIZF), a transcriptional repressor,[52,53] was the strongest coefficient associated with SCP1-promoter-biased activity. In addition to the annotations associated with *INS*-promoter-biased activity in the reduced set, we found that several GATA family TFs and SOX family member SRY motifs were strongly predictive of fragments' bias toward the *INS* promoter, as was the motif for NKX6.1, another key pancreatic β cell identity TF. Similar to *HNF1A*/*HNF1B*, coding variants in *NKX6-1* have more recently been explored as very rare causes of MODY (<1%),[54,55] and T2D-associated variants are also enriched in NKX6.1 motifs.[2,56,57]

We applied the same approach to define features associated with upstream/downstream position bias (Figures 3C and S5B). Of the significantly associated features, most (32/46) exhibited bias toward upstream-cloned fragments. Features predictive of upstream bias included overlap with open chromatin peaks in pancreatic islets ("atac_seq"), motifs for the transcriptional activator NF-Y (which binds to CCAAT motifs in promoters),[58] and several TFs (e.g., CUX1, ISX, POU family members, and PAX family

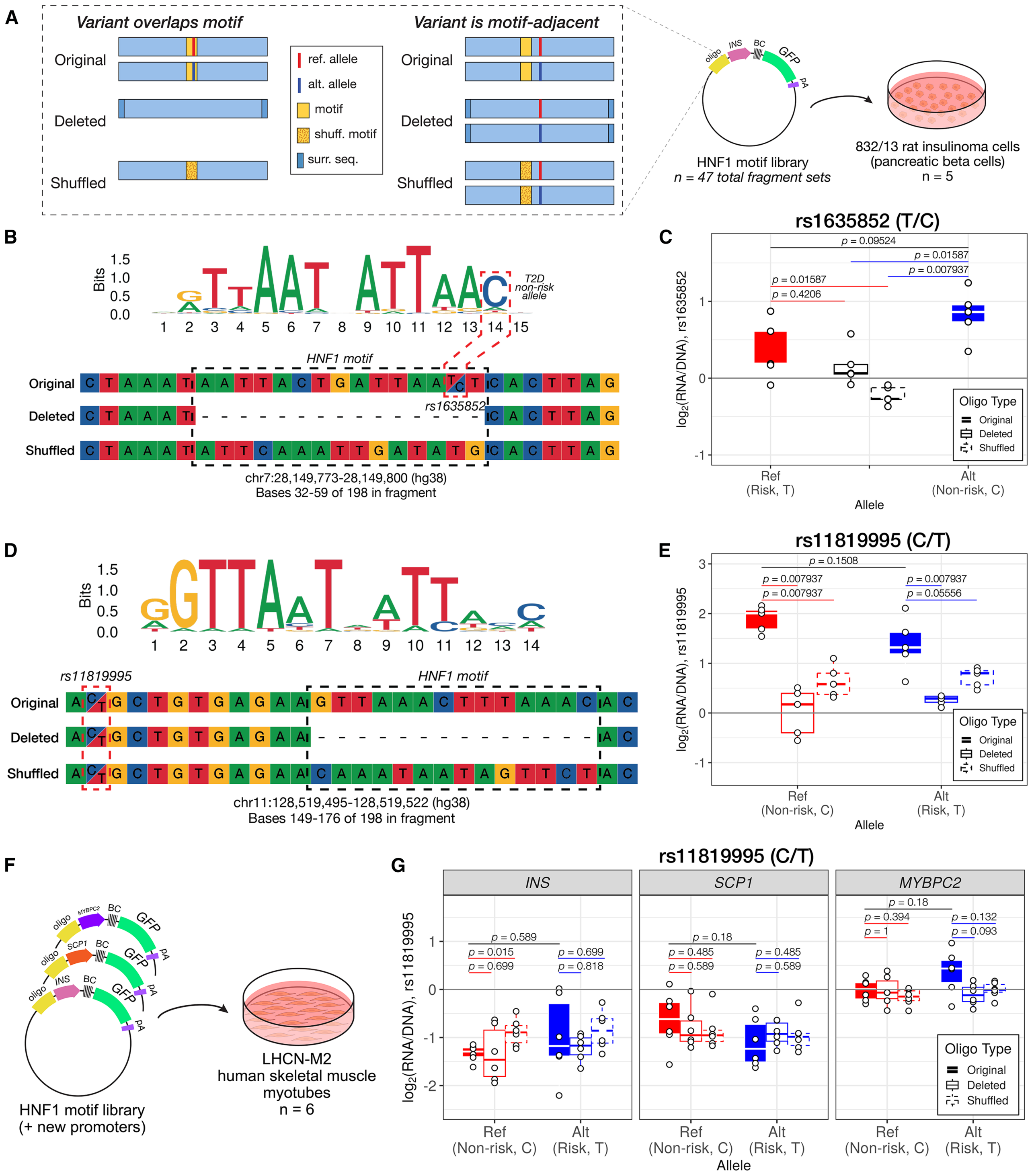

Figure 4. HNF1 transcription factor motifs contribute to enhancer activity near selected T2D-associated variants

(A) For 47 HNF1-motif-overlapping fragments with significant *INS* promoter-bias effects on activity, we designed three versions: original (motif intact), deleted (motif removed and sequence adjusted), and shuffled (dinucleotide-shuffled motif). When the tested variant was adjacent to the motif, we synthesized both reference and alternative alleles for each version. For variants directly overlapping the motif, we generated only one deletion and one shuffled fragment.

(B) We synthesized four fragments corresponding to the variant rs1635852, which overlaps an HNF1 motif at a high-information-content position. The T2D risk allele (T) disrupts this motif, while the non-risk allele (C) matches the consensus.

(C) Shuffling the motif significantly decreased enhancer activity compared to intact fragments with either the risk T (Wilcoxon rank-sum test $p = 0.016$) or non-risk C ($p = 0.008$) allele. Motif deletion also significantly decreased enhancer activity compared to the non-risk C allele ($p = 0.016$).

(D) For the variant rs11819995, located 11 bp upstream of an HNF1 motif, we synthesized six fragments.

(legend continued on next page)

members) known to bind to promoters of target genes important for developmental processes and fate specification.[59] The strongest predictor of upstream-biased activity was the presence of the “Active_TSS,” an annotation denoting active promoters as defined by ChromHMM.[60] Conversely, many fewer annotations were associated with downstream-biased regulatory activity, though among these included the “typicalEnhancer” ChromHMM state, which overlaps conventional enhancer marks such as p300 or H3K27ac.[61]

Evaluating effects of HNF1 motif perturbations on regulatory activity

To explore the putative interaction between the HNF1 motif within regulatory regions and the *INS* promoter, we designed and constructed a targeted MPRA library (Figures 4A and S3A). We selected corresponding variants in 47 fragments with significant *INS*-promoter-biased activity (FDR < 0.05) containing HNF1 motifs, irrespective of their activity across the two positions. To generate motif-deletion or -shuffled fragments, we first identified the most likely motif position using SNP-aware position weight matrix scanning. For motif-deletion fragments, we removed the matched sequence and added (n motif bp)/2 of the surrounding sequence to each end of the fragment to keep the fragment length constant. For motif-shuffled fragments, we performed a dinucleotide shuffle on the matched motif sequence to disrupt the motif while maintaining the same sequence content. In cases where the variant was adjacent to the HNF1 motif (rather than contained within), we synthesized motif-deletion and motif-shuffled fragments with both alleles. We cloned this set of fragments into an MPRA vector upstream of the *INS* promoter, then delivered this library to 832/13 cells ($n = 5$), as described previously.

Of the 47 fragment sets we synthesized and tested, 39 sets had at least one fragment with significant activity (Z score $p < 0.05$) and were retained for downstream analysis (Table S15). Among these 39 sets, 19 displayed differential activity between at least one motif-intact and one modified (deleted or shuffled) fragment (Wilcoxon rank-sum test $p < 0.05$). Here, we present two representative examples that illustrate variable regulatory activity associated with the HNF1 motif. One such variant was rs1635852, which is found in the first intron of *JAZF1*, a well-characterized transcriptional regulator of the pancreatic β cell response to metabolic stress.[62] This variant falls in a high-information-content position of the HNF1 consensus motif (JASPAR record MA0046.2), where changing from the reference allele (T, risk allele for T2D) to the alternative allele (C, non-risk) is predicted to strengthen the motif (Figure 4B). We observed a modest allelic effect in fragments with intact HNF1 motifs (Wilcoxon rank-sum test $p = 0.09524$), with the effect in the expected direction.[63] We did observe that shuffling the HNF1 motif led to a significant decrease in activity compared with either the risk (Wilcoxon rank-sum test $p = 0.01587$) or non-risk (Figure 4C; Wilcoxon rank-sum test $p = 0.007937$) allele fragments. Deleting the HNF1 motif led to significantly decreased activity compared to the non-risk allele alone (Wilcoxon rank-sum test $p = 0.01587$). We note that the risk allele fragment shows higher variance than any of the other fragments, which may reduce our power to detect subtle differences between this fragment and the HNF1-motif-deletion fragment. To evaluate the extent to which these effects were restricted to the *INS* promoter, we also tested these fragments with the SCP1 promoter. We did not observe similar patterns in differential activity based on HNF1 motif status. There is a nearly significant difference (Wilcoxon rank-sum test $p = 0.05556$) between the oligo with the HNF1 motif deleted compared to the intact, reference allele oligo; however, the oligo with the HNF1 motif deleted displayed high variance in activity (Figure S6A). A previous study reported higher activity for the alternative compared with the reference allele using reporter and DNA affinity assays,[63] consistent with the direction of effect we observed. Interestingly, this study concluded that regulatory activity at this locus was mediated by differential interaction with another key TF expressed in β cells, PDX1. In contrast to the HNF1 motif, the rs1635852 risk T allele is slightly preferred over the non-risk C allele in the PDX1 motif (JASPAR record MA0132.2). Because our scrambled HNF1 motif oligo also partially disrupts the PDX1 motif, this assay may also be detecting changes to PDX1 binding. Our results point to a possible complementary regulatory mechanism at this locus mediated by HNF1A/B. We again observed HNF1-motif-dependent activity at rs11819995, a T2D signal variant identified in several multi-ancestry GWAS.[20,64] This variant is located in the first intron of the canonical *ETS1* transcript and overlaps an alternative transcription start site active in pancreatic islets.[20,65] The 14-bp HNF1 motif matched by position weight matrix scanning (*GTTAAACTTTAAAC*) starts 11 bp downstream of the variant in the fragment sequence (Figure 4D). We did not observe a statistically significant allelic difference in activity between the reference and alternative alleles, most likely due to high variance in the alternative allele

(E) Deletion of the motif significantly decreased enhancer activity for both the reference (C, non-risk) and alternative (T, risk) alleles ($p = 0.008$ for both alleles). Shuffling the motif likewise reduced activity for both alleles ($p = 0.008$ for the reference allele and $p = 0.056$ for the alternative allele).
(F) To assess context-specific effects, we cloned these fragments into MPRA vectors with the SCP1 or skeletal-muscle-specific *MYBPC2* promoter and delivered all three to LHCN-M2 human skeletal muscle myotubes ($n = 6$).
(G) When paired with the *INS* promoter, the shuffled rs11819995-containing fragment showed increased activity relative to the original fragment ($p = 0.015$); however, none of the fragments containing rs11819995 functioned as enhancers in LHCN-M2 myotubes, regardless of promoter context. Overall, their activity is highest when paired with the skeletal-muscle-specific promoter.

fragment (Wilcoxon rank-sum $p = 0.1508$). Deleting the HNF1 motif significantly decreased the regulatory activity of fragments, in the presence of either the reference (C, non-risk allele for T2D) or alternative (T, risk) allele of this variant (Wilcoxon rank-sum test $p = 0.007937$ for both alleles; Figure 4E). Additionally, shuffling the HNF1 motif in fragments with either allele decreased activity in comparison to the original fragment (Wilcoxon rank-sum test $p = 0.007937$ for the reference allele and $p = 0.05556$ for the alternative allele). As with the previously described variant, we tested fragments spanning rs11819995 in a construct with the SCP1 promoter. None of these fragments was differentially active based on whether the motif was intact, deleted, or shuffled (Figure S6B).

To further explore the HNF1 motif-*INS* promoter interaction, we considered two related but distinct questions: (1) does perturbing the HNF1 motif have similar effects when cloned in proximity to a different tissue-specific promoter and (2) is the interaction between the HNF1 motif and *INS* promoter restricted to a pancreatic β cell line? To these ends, we cloned the same library of original, deleted, or shuffled fragments into one of two new promoter contexts: either with the SCP1 promoter or with the promoter for the skeletal-muscle-specific gene *MYBPC2*. We then delivered these two libraries, along with the *INS* promoter MPRA library, to the LHCN-M2 human skeletal muscle myoblast line ($n = 6$) after differentiation to myotubes (Figure 4F). In this line, *MYBPC2* is abundantly expressed, and *INS* is transcriptionally silent.

In the LHCN-M2 cell line, none of the HNF1 motif fragment sets displayed significant enhancer activity ($\log_2$(RNA/DNA) > 0 and FDR < 0.05) when paired with any of the three different promoters (Figure 4G). Additionally, we observed that their activity was modulated by promoter context, where the sequences tended to be more active when cloned with the muscle-active *MYBPC2* promoter versus SCP1 or *INS1*. Finally, we observed only one instance in which disrupting the HNF1 motif led to modulated regulatory activity. Compared to the original fragment, scrambling the HNF1 motif caused a modest increase in enhancer activity when paired with the *INS* promoter (Wilcoxon rank-sum test $p = 0.015$); however, HNF1A and HNF1B are not expressed in skeletal muscle, so the enhancer activity of this fragment is likely driven by a separate TF. In all other cases across all three promoter pairings, we no longer observed any dependence upon the HNF1 motif. Taken together, this indicates HNF1A/B-orchestrated regulatory activity is β cell specific.

Discussion

MPRAs enable large-scale variant-to-function studies of *cis*-regulatory elements. However, MPRA implementations differ in key technical aspects, including the cloning and configuration of the reporter construct, whether it is integrated or episomal, and which promoter it carries. Differences in sequence and cellular context can influence the activity of the cloned fragments[5,6]; however, given these screens' scale, it is not routine practice to examine how each of these factors influences individual fragments' activity or how they may modulate the effects of genetic variation.

In this study, we demonstrate that MPRA construct design choices, specifically promoter sequence and proximity, influence the detection of regulatory activity. We found that for a subset of disease-associated genomic sequences, regulatory activity was sensitive to the proximity and identity of the promoter used, independent of overall activity. We found specific genomic annotations that were associated with these biases and noted that the direction of effect was concordant with those interpretations. For instance, fragments annotated as proximal transcription start sites by ChromHMM tended to be more active when cloned upstream, and conversely, those annotated as enhancers were more active when cloned downstream. Of note, we discovered that sequences preferentially active with the human *INS* promoter were enriched in motifs for the important pancreatic β cell TFs, including HNF1A/B and NKX6.1. In a follow-up MPRA, we demonstrated the necessity of HNF1A/B motifs for the activity of a subset of these fragments and confirmed that the dependence upon those motifs was specific to both the cell type and the paired promoters.

Our observation that some sequences' activity depends upon proximity to a promoter is consistent with many previous reports. Regulatory elements are known to work at a range of distances, including thousands of kb from their targets. Here, we identified that sequences that were more active when placed downstream (distal to) the promoter and reporter gene tended to be enriched for features of typical enhancers, while those that were more active in the upstream position (proximal) contained promoter-like elements, including enrichment for active transcription start sites (TSSs). It is important to note that the MPRA construct we used in this study to explore positional effects is distinct from the STARR-seq vector in that fragments are cloned downstream of the poly(A) signal and thus are not transcribed.[6,66] Therefore, we do not suspect that interaction with RNA-binding proteins or accelerated RNA decay influenced the results observed here. Additionally, all fragments in our library were cloned in the genomic forward orientation, and previous work has demonstrated that fragment orientation can influence activity, particularly for sequences overlapping active TSSs. In a separate study, we examined the orientation dependence of regulatory activity at islet-specific active TSSs,[67] and the strong association between the Active_TSS annotation and upstream-biased activity observed here is consistent with directional transcriptional activity from these promoter-like elements. While not directly examined here, these results suggest that

strandedness is another design feature that may influence the interpretation of MPRA results.

Previous studies using reporter assays have indicated that core promoter sequences display some level of tissue specificity,[68] where observed activity in an MPRA vector is strongly correlated with endogenous transcriptional activity from the same loci. This has also been observed by others working to design minimal synthetic promoters that can function in a context-dependent manner, including with respect to cell type.[69] Together with our work, this suggests that promoter sequences contain a unique syntax that interacts with a cell's regulatory milieu to produce highly tuned gene expression beyond the standard elements common to many promoters (e.g., TATA box, DPE, etc.). The two promoters used in the present study differ substantially in sequence content and length (81 bp for SCP1 and 408 bp for *INS*). The *INS* promoter contains multiple cell-type-specific TF binding sites that enable nimble response to glucose stimulation and was previously delimited through deletion mapping and motif analysis.[41,43] While we observed higher overall activity with the *INS* promoter, the relative contributions of tissue-specific compatibility versus intrinsic promoter strength (in part influenced by length) remain unclear from our data. Selecting suitable promoters for other cell types will require a similar level of inspection.

Though not explicitly considered in the work presented here, the choice of using an episomal or integrated MPRA vector has also been examined previously.[6,26,70] Here, we used an integrated construct to compare activity at a specific locus in the LHCN-M2 human skeletal muscle cell line compared to an electroporated episomal construct in the 832/13 rat β cell line (Figures 4D and 4E), due to technical limitations that make electroporation or transfection of differentiated LHCN-M2 cells unfeasible. While we see differences in regulatory activity across the two lines, we believe this is a biological distinction, not a methodological one. An additional source of concern for episomal MPRAs is the preferential use of the bacterial origin of replication (ORI) over the SCP1 promoter.[6,71] Consequently, researchers have advised using other synthetic promoters (e.g., minP) or ORI-less versions of the MPRA backbone to avoid this issue. Given the systematic discrepancies measured between the two methods in the studies referenced above, this design choice may also require careful forethought.

Although our initial MPRA library contained both reference and alternative alleles across a large set of T2D-associated variants in three positional configurations, challenges arose when generating the sequencing libraries to perform barcode-fragment pairing. This was largely driven by inefficiency introduced by the large distance (∼1.5–1.7 kb) between fragments and barcodes for the upstream configurations. This, combined with the use of a conservative association pipeline, limited the final library complexity below what is needed for sensitive detection of allelic effects or systematic comparison across variant positions, which are often subtle. We therefore focused our analyses on fragment-level activity across position and promoter contexts. We compared broad Chrom HMM-designated β cell regulatory annotations (enhancer, promoter, and other) for the subset of 11,656 fragments compared against the full set of 79,356 fragments. We observed a slight increase in the proportions of enhancers (31% in the smaller set versus 26% in the full set) and promoters (9% versus 7%) and a slight decrease in the proportions of any other annotations (60% versus 66%). This suggests that, despite the pairing limitations, the smaller set of fragments is representative of the full library.

The findings from the larger library described here complement prior allele-focused MPRAs, such as one published in 2021 by Khetan and colleagues[2] that characterized over 2,000 T2D-associated variants. Given that the number of T2D-associated genomic loci has more than quadrupled since these libraries were constructed,[20,64,72] it is a high priority to develop new MPRA libraries to profile the expanded set of loci, evaluate all linked variants including indels and rare variants, and to methodically evaluate how MPRA design (e.g., the features explored here or variant position within test fragments) impacts the ability to discover allelic bias in activity.

In summary, our comparison of MPRA design parameters reveals that both promoter choice and fragment position have measurable impacts on regulatory activity and likely vary across different fragments. Though it takes consideration to select appropriate promoters for a cell type of interest, we show that for pancreatic islet β cells, using the *INS* promoter was sufficient to capture regulatory activity at many T2D-associated loci, especially those overlapping relevant β cell TFs. Given the inherent sparsity of MPRA datasets, employing these libraries across a variety of design configurations and cell types will enhance our ability to understand regulatory processes that are disrupted in complex diseases.

Data and code availability

All processed sequencing data generated in this study have been submitted to the NCBI Gene Expression Omnibus (GEO) under accession numbers GEO: GSE279057 (first library), GSE279071 (second library), and GSE247455 (LHCN-M2 data). Corresponding raw sequencing data are available at the NCBI Sequencing Read Archive (SRA): PRJNA1142481 and PRJNA1142558. Complete results files are available as part of this manuscript as Tables S1, S2, S3, S4, S5, S6, S7, S8, S9, S10, S11, S12, S13, S14, and S15. Custom scripts used to preprocess and analyze data from the second MPRA library and other auxiliary analyses are available at Github: https://github.com/adelaidetovar/modular-t2d-mpra.

Acknowledgments

The authors would like to acknowledge members of the Kitzman and Parker labs (University of Michigan) for their critical feedback. The authors would also like to thank the University of

Michigan Viral Vector Core for producing lentivirus and the University of Michigan Advanced Genomics Core for sequencing services. The authors acknowledge support from the National Institute of Diabetes and Digestive and Kidney Diseases, grants 1UM1DK126185-01 (S.C.J.P.), R01 DK117960 (S.C.J.P.), Opportunity Pool Funding (A.T., S.C.J.P., and J.O.K.), and T32DK101357 (A.T.); the National Institute of General Medical Studies R35GM153286 (J.O.K.); the National Human Genome Research Institute K99HG013676 (A.T.); and the Burroughs Wellcome Fund Postdoctoral Diversity Enrichment Fellowship (A.T.).

Author contributions

Conceptualization, A.T., Y.K., J.O.K., and S.C.J.P.; fragment design and MPRA library cloning, Y.K., A.T., and K.N.; data processing and analysis, A.T., Y.K., and M.B., with contributions from A.V. A.T. wrote the manuscript with input from and editing by J.O.K. and S.C.J.P.

Declaration of interests

J.O.K. serves as a scientific advisor to MyOme, Inc.

Supplemental information

Supplemental information can be found online at https://doi.org/10.1016/j.xhgg.2026.100606.

Web resources

GEO, https://www.ncbi.nlm.nih.gov/gds
Github, https://github.com/adelaidetovar/modular-t2d-mpra
SRA, https://www.ncbi.nlm.nih.gov/sra

Received: November 23, 2025
Accepted: April 2, 2026

References

1. Watanabe, K., Stringer, S., Frei, O., Umićević Mirkov, M., de Leeuw, C., Polderman, T.J.C., van der Sluis, S., Andreassen, O.A., Neale, B.M., and Posthuma, D. (2019). A global overview of pleiotropy and genetic architecture in complex traits. Nat. Genet. *51*, 1339–1348.
2. Khetan, S., Kales, S., Kursawe, R., Jillette, A., Ulirsch, J.C., Reilly, S.K., Ucar, D., Tewhey, R., and Stitzel, M.L. (2021). Functional characterization of T2D-associated SNP effects on baseline and ER stress-responsive β cell transcriptional activation. Nat. Commun. *12*, 5242.
3. Tewhey, R., Kotliar, D., Park, D.S., Liu, B., Winnicki, S., Reilly, S.K., Andersen, K.G., Mikkelsen, T.S., Lander, E.S., Schaffner, S.F., and Sabeti, P.C. (2016). Direct Identification of Hundreds of Expression-Modulating Variants using a Multiplexed Reporter Assay. Cell *165*, 1519–1529.
4. Arnold, C.D., Gerlach, D., Stelzer, C., Boryń, Ł.M., Rath, M., and Stark, A. (2013). Genome-wide quantitative enhancer activity maps identified by STARR-seq. Science *339*, 1074–1077.
5. Bergman, D.T., Jones, T.R., Liu, V., Ray, J., Jagoda, E., Siraj, L., Kang, H.Y., Nasser, J., Kane, M., Rios, A., et al. (2022). Compatibility rules of human enhancer and promoter sequences. Nature *607*, 176–184.
6. Klein, J.C., Agarwal, V., Inoue, F., Keith, A., Martin, B., Kircher, M., Ahituv, N., and Shendure, J. (2020). A systematic evaluation of the design and context dependencies of massively parallel reporter assays. Nat. Methods *17*, 1083–1091.
7. Banerji, J., Rusconi, S., and Schaffner, W. (1981). Expression of a beta-globin gene is enhanced by remote SV40 DNA sequences. Cell *27*, 299–308.
8. Ulirsch, J.C., Nandakumar, S.K., Wang, L., Giani, F.C., Zhang, X., Rogov, P., Melnikov, A., McDonel, P., Do, R., Mikkelsen, T.S., and Sankaran, V.G. (2016). Systematic Functional Dissection of Common Genetic Variation Affecting Red Blood Cell Traits. Cell *165*, 1530–1545.
9. Choi, J., Zhang, T., Vu, A., Ablain, J., Makowski, M.M., Colli, L.M., Xu, M., Hennessey, R.C., Yin, J., Rothschild, H., et al. (2020). Massively parallel reporter assays of melanoma risk variants identify MX2 as a gene promoting melanoma. Nat. Commun. *11*, 2718.
10. Lu, X., Chen, X., Forney, C., Donmez, O., Miller, D., Parameswaran, S., Hong, T., Huang, Y., Pujato, M., Cazares, T., et al. (2021). Global discovery of lupus genetic risk variant allelic enhancer activity. Nat. Commun. *12*, 1611.
11. Myint, L., Wang, R., Boukas, L., Hansen, K.D., Goff, L.A., and Avramopoulos, D. (2020). A screen of 1,049 schizophrenia and 30 Alzheimer's-associated variants for regulatory potential. Am. J. Med. Genet. B Neuropsychiatr. Genet. *183*, 61–73.
12. McAfee, J.C., Lee, S., Lee, J., Bell, J.L., Krupa, O., Davis, J., Insigne, K., Bond, M.L., Zhao, N., Boyle, A.P., et al. (2023). Systematic investigation of allelic regulatory activity of schizophrenia-associated common variants. Cell Genom. *3*, 100404.
13. McAfee, J.C., Bell, J.L., Krupa, O., Matoba, N., Stein, J.L., and Won, H. (2022). Focus on your locus with a massively parallel reporter assay. J. Neurodev. Disord. *14*, 50.
14. Melnikov, A., Murugan, A., Zhang, X., Tesileanu, T., Wang, L., Rogov, P., Feizi, S., Gnirke, A., Callan, C.G., Jr., Kinney, J.B., et al. (2012). Systematic dissection and optimization of inducible enhancers in human cells using a massively parallel reporter assay. Nat. Biotechnol. *30*, 271–277.
15. Patwardhan, R.P., Hiatt, J.B., Witten, D.M., Kim, M.J., Smith, R.P., May, D., Lee, C., Andrie, J.M., Lee, S.-I., Cooper, G.M., et al. (2012). Massively parallel functional dissection of mammalian enhancers in vivo. Nat. Biotechnol. *30*, 265–270.
16. Zabidi, M.A., Arnold, C.D., Schernhuber, K., Pagani, M., Rath, M., Frank, O., and Stark, A. (2015). Enhancer-core-promoter specificity separates developmental and housekeeping gene regulation. Nature *518*, 556–559.
17. Martinez-Ara, M., Comoglio, F., van Arensbergen, J., and van Steensel, B. (2022). Systematic analysis of intrinsic enhancer-promoter compatibility in the mouse genome. Mol. Cell *82*, 2519–2531.e6.
18. Hong, C.K.Y., and Cohen, B.A. (2022). Genomic environments scale the activities of diverse core promoters. Genome Res. *32*, 85–96.
19. Sahu, B., Hartonen, T., Pihlajamaa, P., Wei, B., Dave, K., Zhu, F., Kaasinen, E., Lidschreiber, K., Lidschreiber, M., Daub, C.O., et al. (2022). Sequence determinants of human gene regulatory elements. Nat. Genet. *54*, 283–294.
20. Mahajan, A., Spracklen, C.N., Zhang, W., Ng, M.C.Y., Petty, L.E., Kitajima, H., Yu, G.Z., Rüeger, S., Speidel, L., Kim, Y.J., et al. (2022). Multi-ancestry genetic study of type 2 diabetes highlights the power of diverse populations for discovery and translation. Nat. Genet. *54*, 560–572.

21. Chen, J., Spracklen, C.N., Marenne, G., Varshney, A., Corbin, L.J., Luan, J., Willems, S.M., Wu, Y., Zhang, X., Horikoshi, M., et al. (2021). The trans-ancestral genomic architecture of glycemic traits. Nat. Genet. *53*, 840–860.
22. Varshney, A., Kyono, Y., Elangovan, V.R., Wang, C., Erdos, M.R., Narisu, N., Albanus, R.D., Orchard, P., Stitzel, M.L., Collins, F.S., et al. (2021). A transcription start site map in human pancreatic islets reveals functional regulatory signatures. Diabetes *70*, 1581–1591. https://doi.org/10.2337/db20-1087.
23. Sack, L.M., Davoli, T., Xu, Q., Li, M.Z., and Elledge, S.J. (2016). Sources of error in mammalian genetic screens. G3 (Bethesda) *6*, 2781–2790.
24. Hill, A.J., McFaline-Figueroa, J.L., Starita, L.M., Gasperini, M.J., Matreyek, K.A., Packer, J., Jackson, D., Shendure, J., and Trapnell, C. (2018). On the design of CRISPR-based single-cell molecular screens. Nat. Methods *15*, 271–274.
25. Gordon, M.G., Inoue, F., Martin, B., Schubach, M., Agarwal, V., Whalen, S., Feng, S., Zhao, J., Ashuach, T., Ziffra, R., et al. (2020). lentiMPRA and MPRAflow for high-throughput functional characterization of gene regulatory elements. Nat. Protoc. *15*, 2387–2412.
26. Inoue, F., Kircher, M., Martin, B., Cooper, G.M., Witten, D.M., McManus, M.T., Ahituv, N., and Shendure, J. (2017). A systematic comparison reveals substantial differences in chromosomal versus episomal encoding of enhancer activity. Genome Res. *27*, 38–52.
27. Gergics, P., Smith, C., Bando, H., Jorge, A.A.L., Rockstroh-Lippold, D., Vishnopolska, S.A., Castinetti, F., Maksutova, M., Carvalho, L.R.S., Hoppmann, J., et al. (2021). High-throughput splicing assays identify missense and silent splice-disruptive POU1F1 variants underlying pituitary hormone deficiency. Am. J. Hum. Genet. *108*, 1526–1539.
28. Zorita, E., Cuscó, P., and Filion, G.J. (2015). Starcode: sequence clustering based on all-pairs search. Bioinformatics *31*, 1913–1919.
29. Li, H., and Durbin, R. (2009). Fast and accurate short read alignment with Burrows-Wheeler transform. Bioinformatics *25*, 1754–1760.
30. Garrison, E., and Marth, G. (2012). Haplotype-based variant detection from short-read sequencing. arXiv. https://doi.org/10.48550/arXiv.1207.3907.
31. Martin, M. (2011). Cutadapt removes adapter sequences from high-throughput sequencing reads. EMBnet J. *17*, 10–12.
32. Smith, T., Heger, A., and Sudbery, I. (2017). UMI-tools: modeling sequencing errors in Unique Molecular Identifiers to improve quantification accuracy. Genome Res. *27*, 491–499.
33. Ashuach, T., Fischer, D.S., Kreimer, A., Ahituv, N., Theis, F.J., and Yosef, N. (2019). MPRAnalyze: statistical framework for massively parallel reporter assays. Genome Biol. *20*, 183.
34. Tibshirani, R. (1996). Regression shrinkage and selection via the lasso. Journal of the royal statistical society series b-methodological *58*, 267–288.
35. D'Oliveira Albanus, R., Kyono, Y., Hensley, J., Varshney, A., Orchard, P., Kitzman, J.O., and Parker, S.C.J. (2021). Chromatin information content landscapes inform transcription factor and DNA interactions. Nat. Commun. *12*, 1307.
36. McCaw, Z.R., Lane, J.M., Saxena, R., Redline, S., and Lin, X. (2020). Operating characteristics of the rank-based inverse normal transformation for quantitative trait analysis in genome-wide association studies. Biometrics *76*, 1262–1272.
37. Friedman, J., Hastie, T., and Tibshirani, R. (2010). Regularization Paths for Generalized Linear Models via Coordinate Descent. J. Stat. Softw. *33*, 1–22.
38. Mahajan, A., Taliun, D., Thurner, M., Robertson, N.R., Torres, J.M., Rayner, N.W., Payne, A.J., Steinthorsdottir, V., Scott, R.A., Grarup, N., et al. (2018). Fine-mapping type 2 diabetes loci to single-variant resolution using high-density imputation and islet-specific epigenome maps. Nat. Genet. *50*, 1505–1513.
39. Spracklen, C.N., Horikoshi, M., Kim, Y.J., Lin, K., Bragg, F., Moon, S., Suzuki, K., Tam, C.H.T., Tabara, Y., Kwak, S.-H., et al. (2020). Identification of type 2 diabetes loci in 433,540 East Asian individuals. Nature *582*, 240–245.
40. Juven-Gershon, T., Cheng, S., and Kadonaga, J.T. (2006). Rational design of a super core promoter that enhances gene expression. Nat. Methods *3*, 917–922.
41. German, M., Ashcroft, S., Docherty, K., Edlund, H., Edlund, T., Goodison, S., Imura, H., Kennedy, G., Madsen, O., and Melloul, D. (1995). The insulin gene promoter. A simplified nomenclature. Diabetes *44*, 1002–1004.
42. Chandra, V., Albagli-Curiel, O., Hastoy, B., Piccand, J., Randriamampita, C., Vaillant, E., Cavé, H., Busiah, K., Froguel, P., Vaxillaire, M., et al. (2014). RFX6 regulates insulin secretion by modulating Ca2+ homeostasis in human β cells. Cell Rep. *9*, 2206–2218.
43. Melloul, D., Marshak, S., and Cerasi, E. (2002). Regulation of insulin gene transcription. Diabetologia *45*, 309–326.
44. Nkonge, K.M., Nkonge, D.K., and Nkonge, T.N. (2020). The epidemiology, molecular pathogenesis, diagnosis, and treatment of maturity-onset diabetes of the young (MODY). Clin. Diabetes Endocrinol. *6*, 20.
45. Zhang, H., Colclough, K., Gloyn, A.L., and Pollin, T.I. (2021). Monogenic diabetes: a gateway to precision medicine in diabetes. J. Clin. Investig. *131*, e142244. https://doi.org/10.1172/JCI142244.
46. Quang, D.X., Erdos, M.R., Parker, S.C.J., and Collins, F.S. (2015). Motif signatures in stretch enhancers are enriched for disease-associated genetic variants. Epigenetics Chromatin *8*, 23.
47. Okita, K., Yang, Q., Yamagata, K., Hangenfeldt, K.A., Miyagawa, J., Kajimoto, Y., Nakajima, H., Namba, M., Wollheim, C.B., Hanafusa, T., and Matsuzawa, Y. (1999). Human insulin gene is a target gene of hepatocyte nuclear factor-1alpha (HNF-1alpha) and HNF-1beta. Biochem. Biophys. Res. Commun. *263*, 566–569.
48. Párrizas, M., Maestro, M.A., Boj, S.F., Paniagua, A., Casamitjana, R., Gomis, R., Rivera, F., and Ferrer, J. (2001). Hepatic nuclear factor 1-alpha directs nucleosomal hyperacetylation to its tissue-specific transcriptional targets. Mol. Cell Biol. *21*, 3234–3243.
49. Emens, L.A., Landers, D.W., and Moss, L.G. (1992). Hepatocyte nuclear factor 1 alpha is expressed in a hamster insulinoma line and transactivates the rat insulin I gene. Proc. Natl. Acad. Sci. USA *89*, 7300–7304.
50. Pugh, B.F., and Tjian, R. (1990). Mechanism of transcriptional activation by Sp1: evidence for coactivators. Cell *61*, 1187–1197.
51. Louder, R.K., He, Y., López-Blanco, J.R., Fang, J., Chacón, P., and Nogales, E. (2016). Structure of promoter-bound TFIID and model of human pre-initiation complex assembly. Nature *531*, 604–609.

52. Sekimata, M., and Homma, Y. (2004). Sequence-specific transcriptional repression by an MBD2-interacting zinc finger protein MIZF. Nucleic Acids Res. *32*, 590–597.
53. Sekimata, M., Takahashi, A., Murakami-Sekimata, A., and Homma, Y. (2001). Involvement of a novel zinc finger protein, MIZF, in transcriptional repression by interacting with a methyl-CpG-binding protein, MBD2. J. Biol. Chem. *276*, 42632–42638.
54. Mohan, V., Radha, V., Nguyen, T.T., Stawiski, E.W., Pahuja, K.B., Goldstein, L.D., Tom, J., Anjana, R.M., Kong-Beltran, M., Bhangale, T., et al. (2018). Comprehensive genomic analysis identifies pathogenic variants in maturity-onset diabetes of the young (MODY) patients in South India. BMC Med. Genet. *19*, 22.
55. Taylor, B.L., Liu, F.-F., and Sander, M. (2013). Nkx6.1 is essential for maintaining the functional state of pancreatic beta cells. Cell Rep. *4*, 1262–1275.
56. Chiou, J., Zeng, C., Cheng, Z., Han, J.Y., Schlichting, M., Miller, M., Mendez, R., Huang, S., Wang, J., Sui, Y., et al. (2021). Single-cell chromatin accessibility identifies pancreatic islet cell type- and state-specific regulatory programs of diabetes risk. Nat. Genet. *53*, 455–466.
57. Khetan, S., Kursawe, R., Youn, A., Lawlor, N., Jillette, A., Marquez, E.J., Ucar, D., and Stitzel, M.L. (2018). Type 2 Diabetes-Associated Genetic Variants Regulate Chromatin Accessibility in Human Islets. Diabetes *67*, 2466–2477.
58. Dolfini, D., Gatta, R., and Mantovani, R. (2012). NF-Y and the transcriptional activation of CCAAT promoters. Crit. Rev. Biochem. Mol. Biol. *47*, 29–49.
59. Vadnais, C., Awan, A.A., Harada, R., Clermont, P.-L., Leduy, L., Bérubé, G., and Nepveu, A. (2013). Long-range transcriptional regulation by the p110 CUX1 homeodomain protein on the ENCODE array. BMC Genom. *14*, 258.
60. Ernst, J., and Kellis, M. (2012). ChromHMM: automating chromatin-state discovery and characterization. Nat. Methods *9*, 215–216.
61. Vahedi, G., Kanno, Y., Furumoto, Y., Jiang, K., Parker, S.C.J., Erdos, M.R., Davis, S.R., Roychoudhuri, R., Restifo, N.P., Gadina, M., et al. (2015). Super-enhancers delineate disease-associated regulatory nodes in T cells. Nature *520*, 558–562.
62. Kobiita, A., Godbersen, S., Araldi, E., Ghoshdastider, U., Schmid, M.W., Spinas, G., Moch, H., and Stoffel, M. (2020). The Diabetes Gene JAZF1 Is Essential for the Homeostatic Control of Ribosome Biogenesis and Function in Metabolic Stress. Cell Rep. *32*, 107846.
63. Fogarty, M.P., Panhuis, T.M., Vadlamudi, S., Buchkovich, M.L., and Mohlke, K.L. (2013). Allele-specific transcriptional activity at type 2 diabetes-associated single nucleotide polymorphisms in regions of pancreatic islet open chromatin at the JAZF1 locus. Diabetes *62*, 1756–1762.
64. Suzuki, K., Hatzikotoulas, K., Southam, L., Taylor, H.J., Yin, X., Lorenz, K.M., Mandla, R., Huerta-Chagoya, A., Melloni, G.E.M., Kanoni, S., et al. (2024). Genetic drivers of heterogeneity in type 2 diabetes pathophysiology. Nature *627*, 347–357.
65. Varshney, A., Scott, L.J., Welch, R.P., Erdos, M.R., Chines, P.S., Narisu, N., Albanus, R.D., Orchard, P., Wolford, B.N., Kursawe, R., et al. (2017). Genetic regulatory signatures underlying islet gene expression and type 2 diabetes. Proc. Natl. Acad. Sci. USA *114*, 2301–2306.
66. Das, M., Hossain, A., Banerjee, D., Praul, C.A., and Girirajan, S. (2023). Challenges and considerations for reproducibility of STARR-seq assays. Genome Res. *33*, 479–495.
67. Bose, M., Tovar, A., Kyono, Y., Varshney, A., Kitzman, J.O., and Parker, S.C.J. (2025). Massively parallel reporter assay reveals promoter-position-and strand-specific effects in transcription start sites. bioRxiv. https://doi.org/10.1101/2025.10.13.659964.
68. Mattioli, K., Volders, P.-J., Gerhardinger, C., Lee, J.C., Maass, P.G., Melé, M., and Rinn, J.L. (2019). High-throughput functional analysis of lncRNA core promoters elucidates rules governing tissue specificity. Genome Res. *29*, 344–355.
69. Zahm, A.M., Owens, W.S., Himes, S.R., Fallon, B.S., Rondem, K.E., Gormick, A.N., Bloom, J.S., Kosuri, S., Chan, H., and English, J.G. (2024). A massively parallel reporter assay library to screen short synthetic promoters in mammalian cells. Nat. Commun. *15*, 10353.
70. Guzman, C., Duttke, S., Zhu, Y., De Arruda Saldanha, C., Downes, N.L., Benner, C., and Heinz, S. (2023). Combining TSS-MPRA and sensitive TSS profile dissimilarity scoring to study the sequence determinants of transcription initiation. Nucleic Acids Res. *51*, e80. https://doi.org/10.1093/nar/gkad562.
71. Muerdter, F., Boryń, Ł.M., Woodfin, A.R., Neumayr, C., Rath, M., Zabidi, M.A., Pagani, M., Haberle, V., Kazmar, T., Catarino, R.R., et al. (2018). Resolving systematic errors in widely used enhancer activity assays in human cells. Nat. Methods *15*, 141–149.
72. Vujkovic, M., Keaton, J.M., Lynch, J.A., Miller, D.R., Zhou, J., Tcheandjieu, C., Huffman, J.E., Assimes, T.L., Lorenz, K., Zhu, X., et al. (2020). Discovery of 318 new risk loci for type 2 diabetes and related vascular outcomes among 1.4 million participants in a multi-ancestry meta-analysis. Nat. Genet. *52*, 680–691.

HGGA, Volume 7

Supplemental information

Using a modular massively parallel reporter assay to discover context-dependent regulatory activity in type 2 diabetes-linked noncoding regions

Adelaide Tovar, Yasuhiro Kyono, Kirsten Nishino, Maya Bose, Arushi Varshney, Stephen C. J. Parker, and Jacob O. Kitzman

Supplementary Figures and Figure Legends

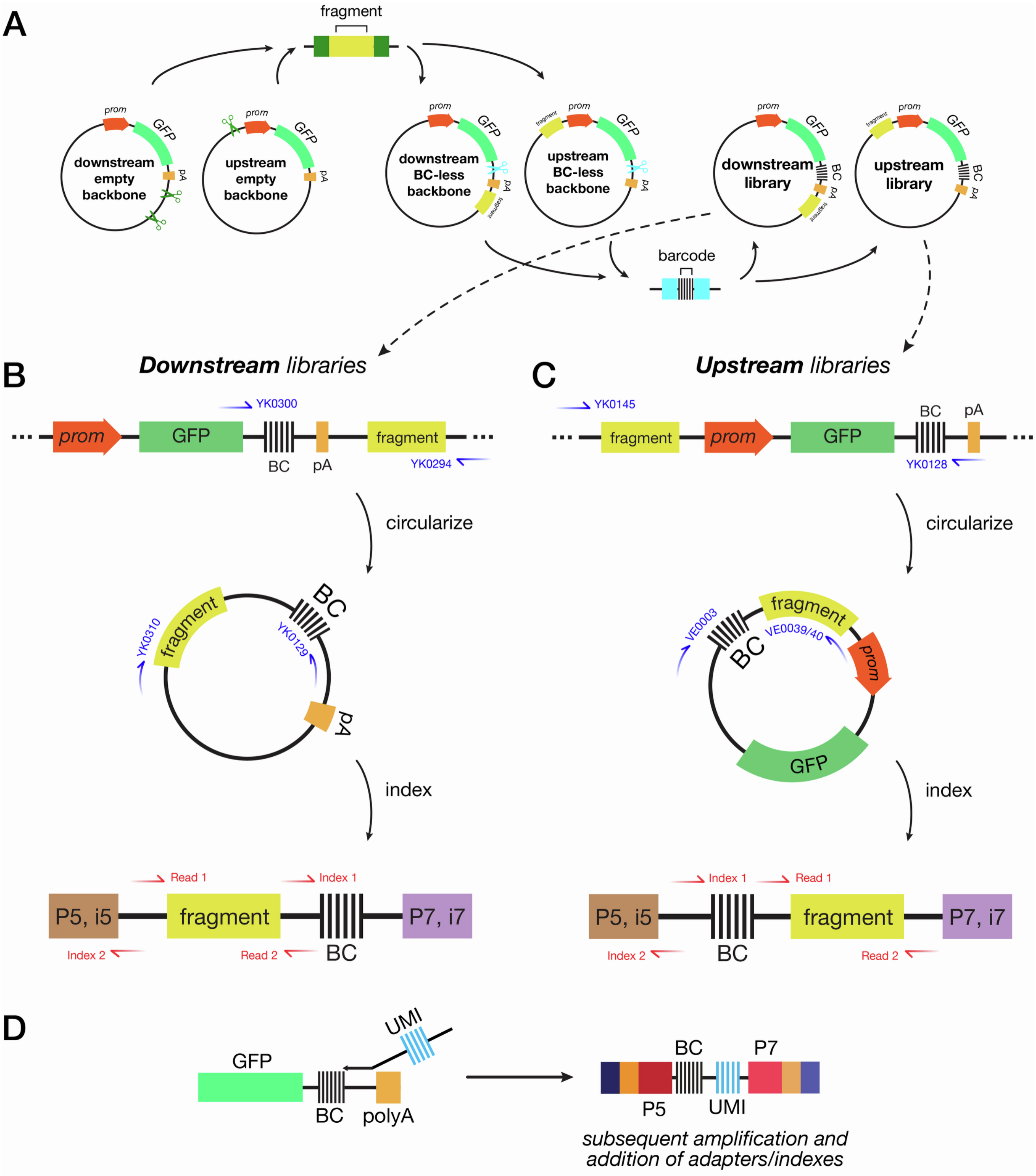

Figure S1. Construction of first MPRA plasmid libraries and barcode sequencing libraries. (A) Construction process for the first MPRA library. We inserted oligo sequences via Gibson assembly into EcoRV- or KpnI-digested (downstream or upstream, respectively) modified STARR-seq backbones that contained either the SCP1 or human insulin (*INS*) promoter. We digested the resulting barcode-less plasmids with PmeI

and inserted barcodes. (B) To perform fragment-barcode pairing for the downstream libraries, we amplified the entire region containing (from 5'-3') the barcode, poly(A) signal, and fragment. We circularized this amplicon and used primers flanking the 5' end of the fragment and 3' end of the barcode. We added sequencing indexes and adapters. We used the index 2 read for sample/library identification, read 1 for the 5' end of the fragment, read 2 for the 3' end of the fragment, and index 1 read for the barcode. **(C)** We performed a similar process as described in **(B)** for the upstream libraries, except the initial amplicon contains (from 5'-3') the fragment, promoter, GFP, and barcode, and the barcode is brought upstream of the fragment after the circularization process. The read layout for the upstream pairing libraries remains the same. **(D)** To construct barcode sequencing libraries using mRNA extracted from cells, we performed reverse transcription using a custom primer containing a unique molecular identifier (UMI) to correct for PCR duplication in sequencing data. Primers were designed separately for each of the two MPRA types to accommodate flanking sequence differences. Subsequently we amplified the cDNA and added sequencing indexes/adapters via PCR.

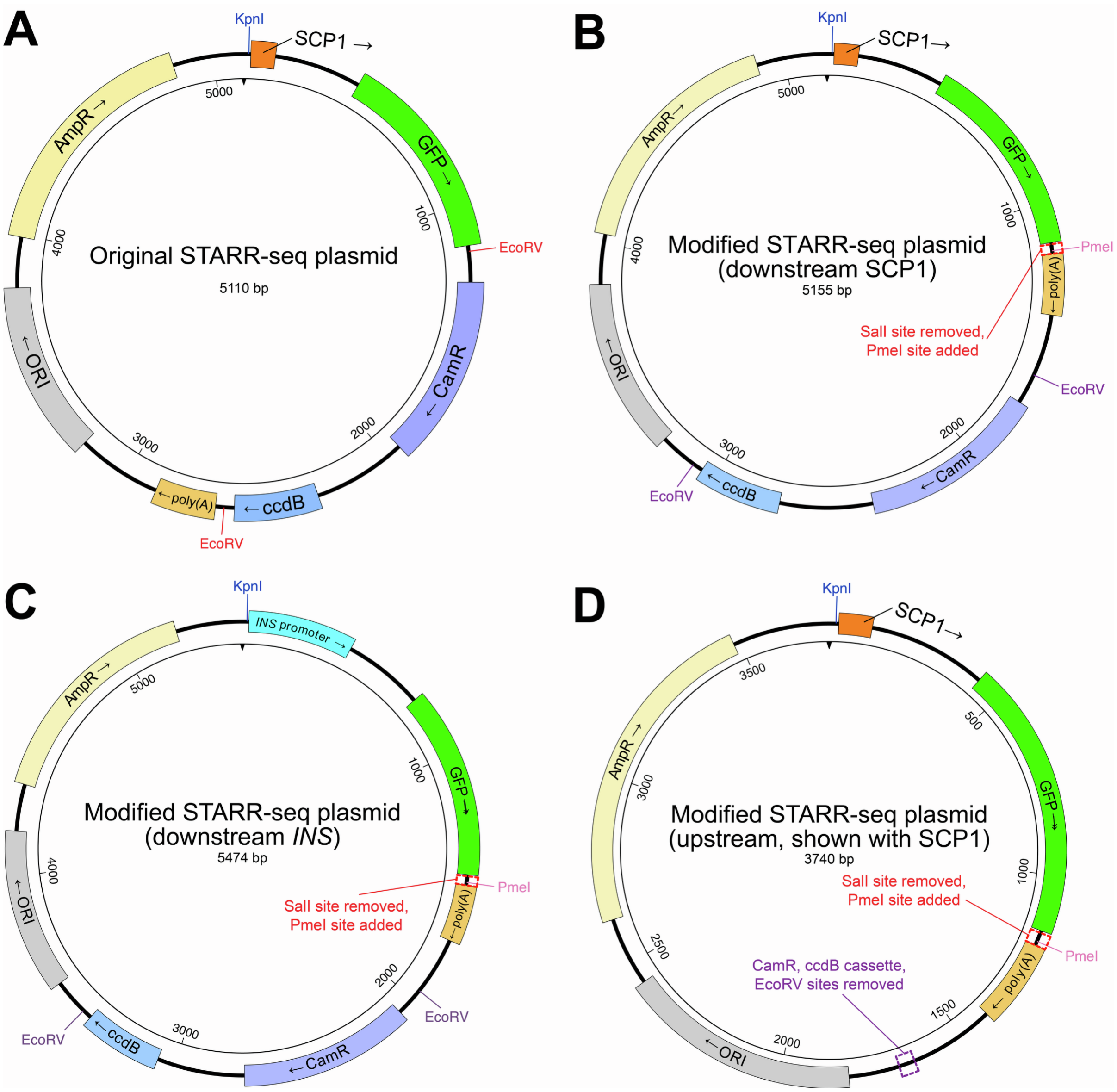

Figure S2. Cloning process to derive modified STARR-seq vectors for the first MPRA library. Arrows next to labels indicate the orientation. **(A)** Simplified diagram for the original STARR-seq vector. **(B)** To generate the downstream SCP1 plasmid, we swapped the position of the ccdB/CamR region and the poly(A) signal while also modifying restriction sites between the 3' end of GFP and the poly(A) signal (removed SalI and added PmeI). Here, the EcoRV restriction sites are used to clone in the fragments. **(C)** We swapped in the *INS* promoter at the SCP1 site to create the downstream *INS* plasmid. **(D)** Finally, to generate the upstream versions of the plasmid we removed the ccdB/CamR region entirely (also removing the EcoRV restriction sites) to bypass the original Gateway system and allow for direct cloning into the vector at the upstream KpnI site.

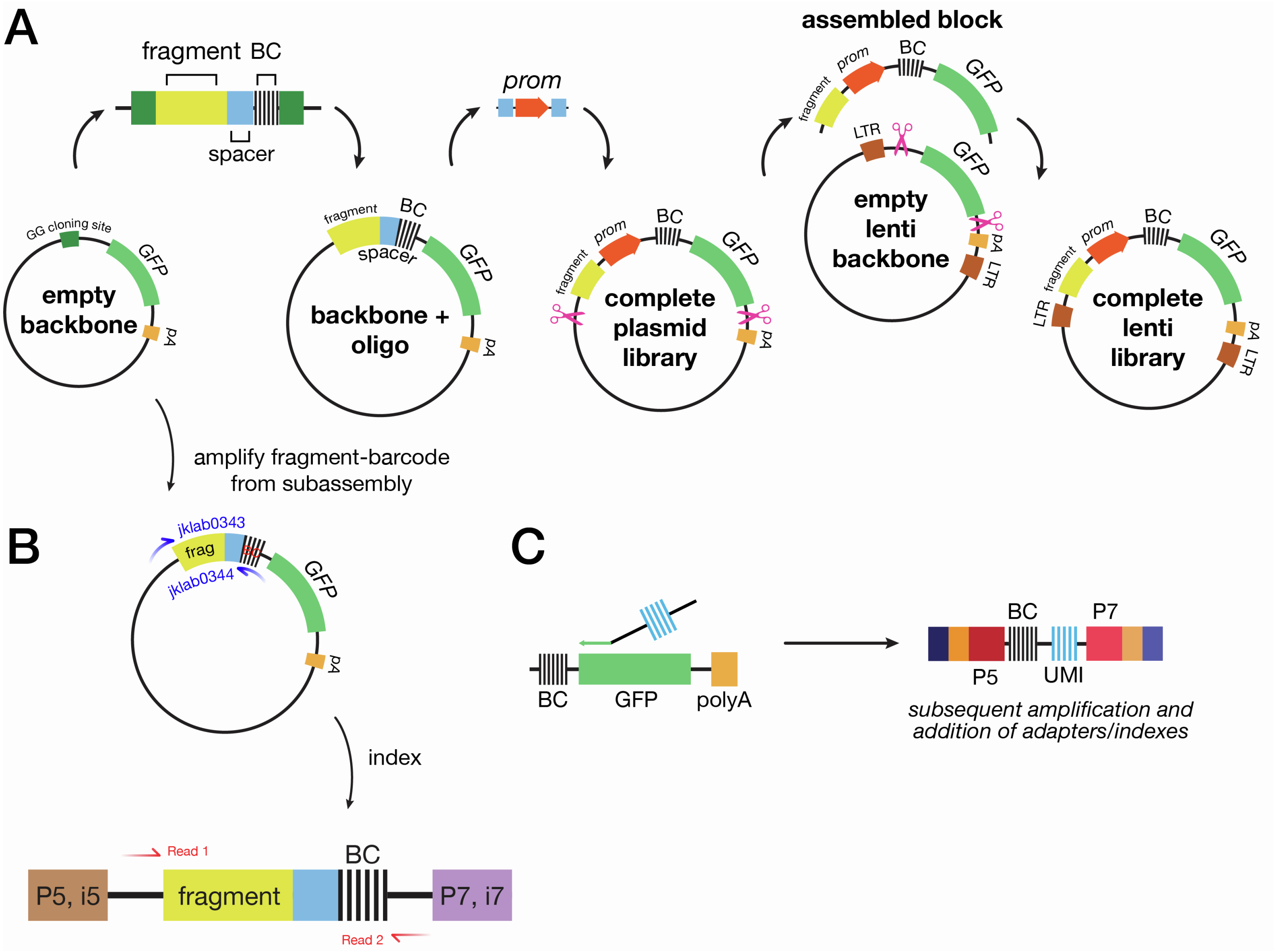

Figure S3. Construction of HNF1 motif-focused MPRA libraries. (A) Construction process for the second MPRA library. We PCR-barcoded oligo sequences and inserted them into the backbone via PaqCI-mediated Golden Gate cloning. We inserted the SCP1 or *INS* promoter in a spacer sequence between the oligo and barcode via BsaI-mediated Golden Gate cloning. **(B)** To construct fragment-barcode pairing libraries, we generated an amplicon spanning the fragment-barcode region of the sub-assembled plasmid. **(C)** Similar to the first MPRA library, we constructed barcode sequencing libraries for the targeted MPRA experiments using a custom primer containing a UMI, this time targeted to the 5' end of GFP.

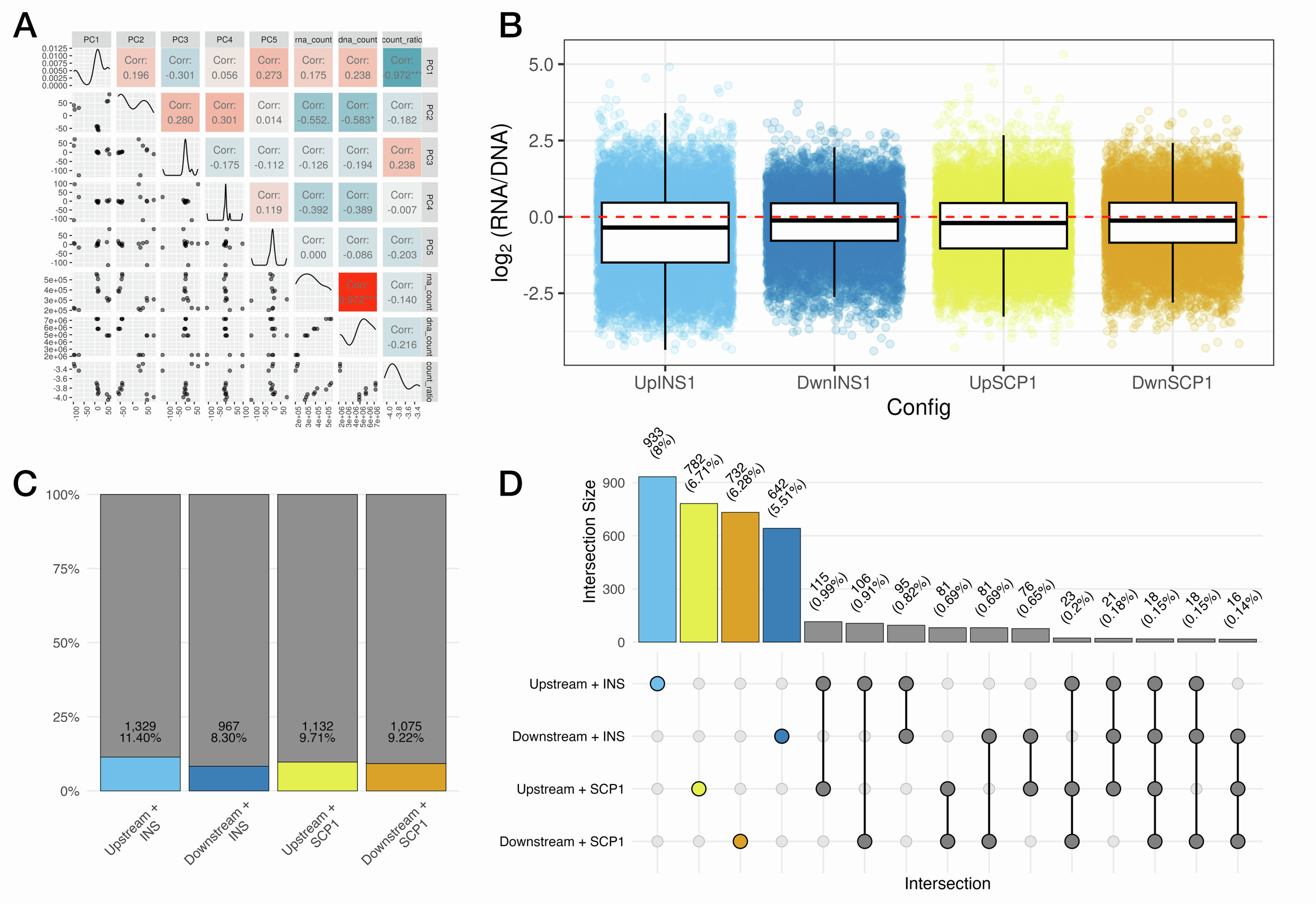

Figure S4. MPRA plasmid configuration modestly influences overall activity of tested fragments and specific sets of significantly active (FDR < 0.05) fragments. (A) Pairwise Spearman correlation heatmap for the first five principal components (from analysis presented in **Figure 2A**), library size-normalized RNA and DNA counts, and ratio between normalized RNA and DNA counts. **(B)** Activity distributions for each of the four configurations shown as the individual activity estimates (average across three replicates). The red dashed line represents a $\log_2$(RNA/DNA) = 0 (i.e., RNA = DNA). (**C)** Stacked barplot showing the number and proportion of fragments that are significantly active (FDR < 0.05) in each plasmid configuration. Percentages are calculated using 11,656 (total number of common fragments) as the denominator. **(D)** UpSet plot displaying intersections (shared and unique) between the sets of fragments that are active in each plasmid configuration. Sets are depicted in the matrix on the bottom, where nodes represent individual configurations (labeled on the left) and edges connect the nodes to form intersections. The bar chart on the top shows the size of each intersection as both the number and proportion of fragments that are significantly active (FDR < 0.05).

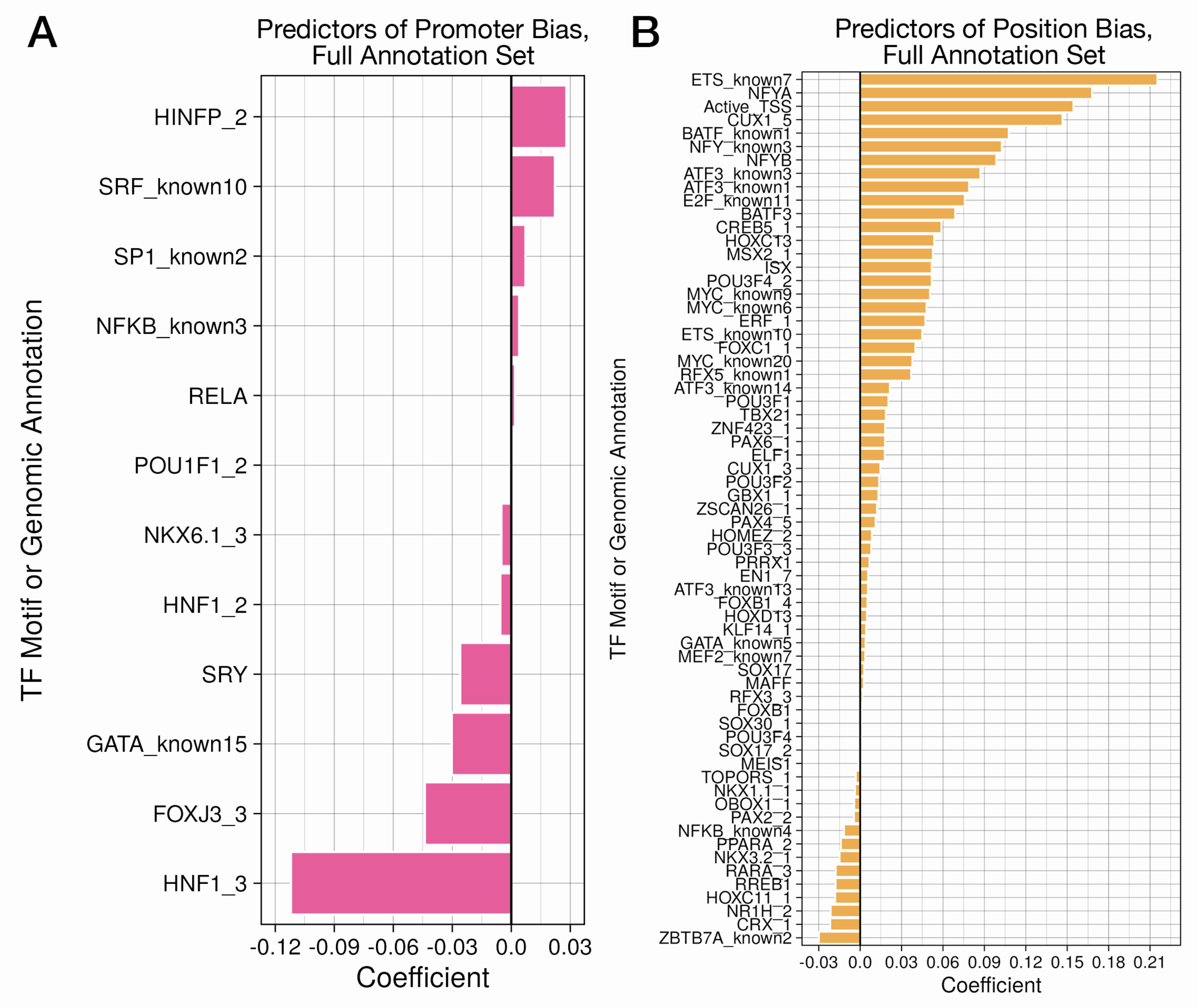

Figure S5. LASSO regression analysis largely identifies the same set of features associated with position or promoter bias when using an expanded set of transcription factor motifs. (A) Full set of significant predictors of promoter bias are displayed as a companion to **Figure 3B**. We identified largely the same set of coefficients. One notable addition is NKX6.1, another beta cell-specific transcription factor. **(B)** Full set of predictors of position bias effects are shown. The ‘activeEnhancer’ chromatin annotation is no longer listed, though ‘X1_Active_TSS’ is still among the strongest predictors of upstream positional bias.

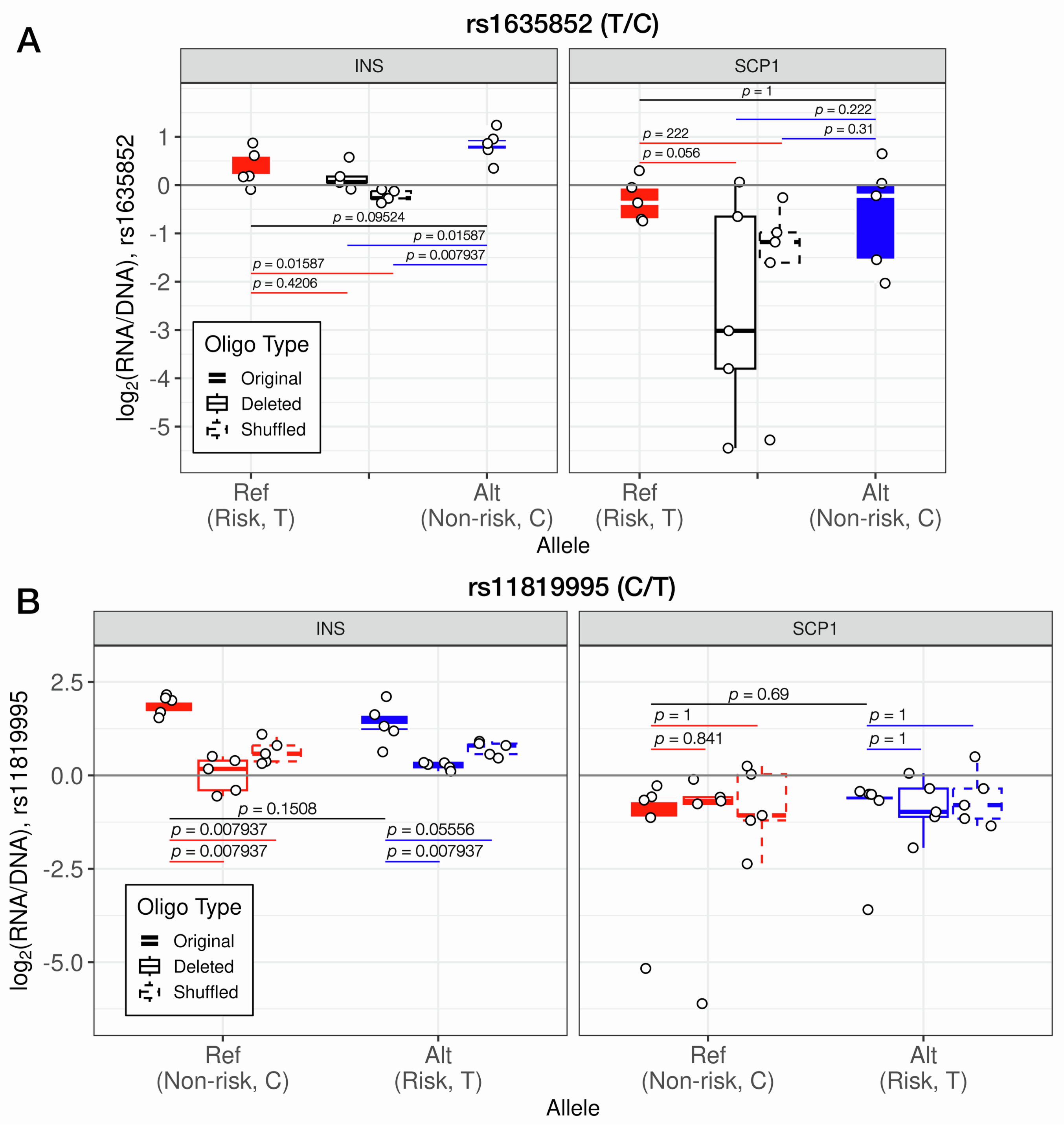

Figure S6. Enhancer activity is minimal for variants near HNF1 transcription factor motifs when paired with the SCP1 promoter. For fragments described in **Figure 4** (reproduced in the left facets), we also examined activity when paired with the synthetic SCP1 housekeeping promoter (displayed in the right facets). **(A)** Manipulating the HNF1 motif overlapping the T2D risk variant rs1635852 does not reproduce the pattern of differential activity seen with the *INS* promoter when paired with the SCP1 promoter, though there is a nearly significant difference in activity between the intact, reference allele oligo and the oligo with the HNF1 motif deleted. We do not observe an allelic difference in activity between the risk (T, reference) and non-risk (C,

alternate) alleles when paired with the SCP1 promoter. **(B)** Similar to the previous variant, none of the fragments for the variant rs11819995 have significant activity when paired with the SCP1 promoter. In contrast to when they're paired with the *INS* promoter, manipulating the HNF1 motif near this variant has no significant effect on activity compared to the original, motif-intact versions of these fragments with the SCP1 promoter (Wilcoxon rank-sum test *p* values reported, n = 5).
